# Bioactive Phenolics from *Medemia argun*: Biological Activities, Molecular Docking, Encapsulation in Alginate–Whey Protein Hydrogel Beads, and Functional Yoghurt Fortification

**DOI:** 10.3390/antiox15091084

**Published:** 2026-08-28

**Authors:** Mohammad S. Mohammad, Sally S. Sakr, Marwa A. Kamel, Amira A. Gamal, Eman S. Abou-Amra, Asmahan A. Ali, Marwa M. El-Said

**Affiliations:** 1 Physics Department, Faculty of Science, Aswan University, Aswan 81528, Egypt; mohamedsaleh@sci.aswu.edu.eg; 2 Department of Food Science and Human Nutrition, College of Agriculture and Food, Qassim University, Buraydah 51452, Saudi Arabia; a.azhari@qu.edu.sa; 3 Environmental Virology Lab, Water Pollution Research Department, Environment and Climate Change Research Institute, National Research Centre, Cairo 12622, Egypt; ma.kamel-ismail@nrc.sci.eg; 4 Chemistry of Natural and Microbial Products Department, Pharmaceutical and Drug Industries Research Institute, National Research Centre, Cairo 12622, Egypt; ag.abdelrahim@nrc.sci.eg; 5 Department of Chemistry, Organic Chemistry, Faculty of Science (Girls), Al-Azhar University, Youssef Abbas Street, Cairo 11754, Egypt; emansadek.59@azhar.edu.eg; 6 Dairy Department, Food Industries and Nutrition Research Institute, National Research Centre, Cairo 12622, Egypt

**Keywords:** *Medemia argun*, phenolic compounds, antioxidant activity, antiviral–antimicrobial potential, molecular docking, hydrogel, functional yoghurt

## Abstract

*Medemia argun* is a rare desert palm rich in phenolic compounds with promising biological activities; however, the instability and poor compatibility of phenols in dairy systems limit their functional application. This study characterized the phenolic composition and biological activities of *M. argun* phenolic extract (MAPE), developed sodium alginate–whey protein concentrate (SAlg/WP) hydrogel beads for phenolic encapsulation, and evaluated their application in fortified yoghurt. HPLC identified quercetin and gallic acid as the predominant phenolic compounds. Antioxidant, antimicrobial, antiviral, and molecular docking analyses were performed, while SAlg/WP–MAPE beads were characterized by encapsulation efficiency analysis, FTIR, and scanning electron microscopy. Fortified yoghurt containing different concentrations of encapsulated MAPE was evaluated for color, syneresis, pH, and antioxidant activity during 14 days of refrigerated storage. MAPE exhibited strong antioxidant and broad-spectrum antimicrobial activities against bacterial and fungal strains, as well as promising antiviral activity against hepatitis A virus (HAV), with an IC_50_ of 104.89 μg/mL and a selectivity index of 1.6. Molecular docking revealed favorable interactions of rutin, chlorogenic acid, and rosmarinic acid with the HAV 3C protease. SAlg/WP beads achieved 78.92% encapsulation efficiency, while FTIR and SEM confirmed successful phenolic entrapment. Encapsulation effectively preserved MAPE functionality and significantly enhanced yoghurt antioxidant activity during storage, demonstrating its potential for functional dairy applications.

## 1. Introduction

Increasing consumer demand for natural, health-promoting foods has accelerated the development of functional foods enriched with bioactive compounds. Functional foods are designed not only to provide basic nutrition, but also to deliver physiological benefits, thereby contributing to disease prevention and overall health promotion. Among the various bioactive constituents incorporated into functional foods, plant-derived phenolic compounds have attracted considerable scientific and industrial interest [1,2]. Phenolic compounds, particularly flavonoids and phenolic acids, are well known for their antioxidant, antimicrobial, anti-inflammatory, and antiviral activities owing to their ability to scavenge free radicals, chelate pro-oxidant metals, and modulate microbial and viral metabolic pathways [3]. Recently, attention to plant-derived phenols as natural antiviral agents against RNA viruses has increased, with compounds such as quercetin, rutin, chlorogenic acid, and rosmarinic acid showing inhibitory effects toward viral replication and protease activity [4,5]. Consequently, plant-derived phenolic compounds have emerged as promising ingredients for developing nutraceuticals and functional foods with enhanced health-promoting properties.

*Medemia argun*, commonly known as argun palm, is a rare desert palm species native to the Nubian Desert regions of Egypt and Sudan. Although the fruits of *M. argun* have traditionally been used in folk medicine [6], scientific investigations concerning their phytochemical composition and biological activities remain limited. Previous studies have demonstrated that *M. argun* fruits contain substantial amounts of bioactive phenolic compounds with antioxidant and antimicrobial properties [7,8]. Desert plants are generally recognized as rich sources of secondary metabolites because exposure to harsh environmental conditions, including drought, high temperature, and oxidative stress, stimulates the accumulation of protective phenolic constituents. Therefore, *M. argun* represents a promising but underexplored source of multifunctional bioactive compounds suitable for nutraceutical and functional food applications.

The remarkable biological activities of plant phenols have spurred extensive research into their mechanisms of action and interactions with molecular targets implicated in human diseases. To better understand the molecular mechanisms underlying these biological activities, molecular docking has become an important computational tool for predicting ligand–target interactions and identifying potential binding modes with enzymes and viral proteins. In particular, molecular docking has been widely applied to investigate the binding affinity of phenolic compounds for biologically relevant targets, providing valuable insights into their antioxidant, antimicrobial, and antiviral potential. Nevertheless, despite the growing interest in plant phenols, limited information is available regarding the interactions of *M. argun* phenolic compounds with biologically relevant molecular targets.

Although hepatitis A virus (HAV) is commonly associated with contaminated fresh produce and raw shellfish, recent studies have reported that dairy products, especially raw milk and soft cheeses, could also be a source for HAV infection [9,10]. Also, dairy products mixed with unheated high-risk ingredients such as frozen berries in yoghurts, pastries, or ice cream could act as secondary vectors for HAV transition within dairy products. Because HAV transmission is mostly via the fecal-oral route, infected personnel handling food during post-processing packaging, cheese-making, or smoothie/ice cream preparation represents a main point of viral introduction into food [11,12].

Although standard pasteurization effectively inactivates HAV, in raw dairy products or minimally heat-treated items, HAV can survive at low temperatures and under acidic conditions (such as fermented dairy environments) for a long time [13]. Hence, natural anti-HAV compounds, including phenolic compounds, such as the polyphenol epigallocatechin-3-gallate, proanthocyanidins, and others, which are reported to exhibit strong virucidal activity against HAV, could serve as functional additives to enhance the safety of dairy products [14,15]. Furthermore, the high content of fats and proteins in dairy products create unique matrix interferences that protect pathogens, but incorporation of plant phenolic compounds as functional ingredients or active edible coatings helps reduces viral persistence in dairy products [16]. Furthermore, natural phenols could act alongside intrinsic dairy peptides and antimicrobial proteins to enhance the microbial safety of raw or minimally processed dairy products [17].

Despite the significant biological potential of plant phenols, their direct incorporation into food systems remains challenging because of their poor stability, susceptibility to oxidation, low bioavailability, and undesirable interactions with food macromolecules, particularly proteins [18]. In dairy systems, direct addition of phenolic compounds may negatively affect protein stability, fermentation behavior, texture, and sensory acceptability due to polyphenol–protein interactions [19]. Furthermore, phenolic compounds are highly sensitive to environmental conditions such as oxygen, light, heat, and processing stresses, which may result in substantial degradation and reduced biological activity during food processing and storage. Therefore, the development of effective encapsulation and delivery systems is essential to improve the stability, protection, controlled release, and functional performance of phenolic compounds in fortified food applications [20].

Encapsulation technology has emerged as an efficient approach to protect sensitive bioactive compounds from environmental degradation while enhancing their stability, bioavailability, and controlled release within food matrices [21]. Among encapsulation materials, sodium alginate has received considerable attention for its biodegradability, biocompatibility, non-toxicity, and mild gelation properties. Moreover, combining sodium alginate with proteins such as whey protein concentrate can improve encapsulation efficiency and structural stability through electrostatic interactions and hydrogen bonding among proteins, polysaccharides, and phenolic compounds [22]. Whey protein is particularly attractive as a wall material because of its high nutritional value, excellent emulsifying properties, and ability to form stable complexes with polyphenols [23]. Previous studies demonstrated that alginate–protein systems effectively enhanced the retention, stability, and controlled release of encapsulated phenols during processing and storage [22,24]. Recently, sodium alginate/collagen hydrolysate beads successfully protected tea polyphenols and maintained their antioxidant activity during storage and thermal processing [25]. However, no studies have investigated the encapsulation of *M. argun* phenols within alginate–whey protein hydrogel systems.

Yoghurt is one of the most widely consumed fermented dairy products worldwide. It is considered an ideal carrier for functional ingredients due to its high nutritional value, digestibility, probiotic properties, and consumer acceptance. In addition to its nutritional benefits, yoghurt consumption has been associated with modulation of the intestinal microbiota, enhancement of the immune response, and improvement of gastrointestinal health [23,26]. Therefore, increasing interest has recently focused on the development of fortified yoghurt products enriched with natural antioxidants and plant-derived bioactive compounds [18]. Incorporating encapsulated phenols into yoghurt systems may improve the stability of bioactive compounds while minimizing undesirable interactions between polyphenols and milk proteins, thereby preserving yoghurt quality and enhancing antioxidant activity during refrigerated storage [27]. Accordingly, encapsulated plant phenols may serve as multifunctional ingredients for developing value-added functional dairy products with improved health-promoting properties.

While the biological activities of plant phenols have been widely studied, there is limited information available on the phenolic compounds found in *M. argun*, their interactions with relevant biological targets, their stabilization through encapsulation with alginate–whey protein hydrogels, and their potential use in functional yoghurt. Therefore, this study aims to characterize the phenolic constituents of *M. argun*, evaluate their biological activities, explore their interactions with selected biological targets using molecular docking, encapsulate the phenolic extract within alginate–whey protein hydrogel beads, and assess the feasibility of using these encapsulated phenols for developing functional yoghurt.

## 2. Materials and Methods

### 2.1. Materials

Dry *M. argun* fruits were collected in October 2025 from argun palm trees in Aswan, Egypt. Dried fruits were sorted and cleaned, then packed in polyethylene bags. The plant specimen was taxonomically identified and deposited in the National Research Centre (NRC) herbarium under voucher number M304. Whey protein concentrate (WP, 80% protein content) was obtained from Davisco Foods International Inc. (Le Sueur, MN, USA). Sodium alginate (SAlg) and calcium chloride (CaCl_2_), 2,2-diphenyl-1-picrylhydrazyl (DPPH) and 2,2′-azinobis (3-ethylbenzothiazoline-6-sulfonic acid) (ABTS) were purchased from Sigma-Aldrich (St. Louis, MO, USA). The tested pathogens were obtained from the biobank of El-Demerdash Hospital. They included *Staphylococcus aureus* (ATCC 25923), *Bacillus cereus* (ATCC 33018), *Bacillus subtilis* (ATCC 6633), *Escherichia coli* (ATCC 8739), *Pseudomonas aeruginosa* (ATCC 14028), *Candida albicans* (ATCC 10231), and *Aspergillus niger* (EM77). Low-fat buffalo milk was supplied by the Animal Production Research Institute, Agriculture Research Center, Dokki, Egypt. Direct Vat Set (DVS) yoghurt starter culture consisted of *Streptococcus thermophilus* and *Lactobacillus delburkii* subsp. *bulgaricus* was obtained from Chr. Hansen Laboratories (Chr. Hansen Holding A/S, 2970 Hoersholm, Denmark). All analytical-grade chemicals, extraction solvents, and standard materials for HPLC analysis (gallic acid, chlorogenic acid, catechin, Methyl gallate, caffeic acid, syringic acid, rutin, ellagic acid, coumaric acid, vanillin, ferulic acid, naringenin, rosmarinic acid, daidzein, quercetin, cinnamic acid, kaempferol and hesperidin) were purchased from Sigma-Aldrich Chemical Company (St. Louis, MO, USA). 

### 2.2. Methods

#### 2.2.1. Preparation of Hydroalcoholic Extract of *M. argun*

*M. argun* phenolic extract (MAPE) was prepared as described in our previous study, with modifications [28]. Fifty grams of *M. argun* was soaked in 250 mL of an ethanol–water mixture (80:20, *v*/*v*) in an amber flask and agitated at 120 rpm for 12 h at room temperature. The extract was centrifuged at 5000× *g* for 15 min (Shanghai Lu Xiangyi Centrifuge Instrument Co., Shanghai, China). To maximize phenolic compound recovery, the extraction was repeated three times, and the resulting supernatants were pooled. Ethanol was subsequently evaporated under reduced pressure at 40 °C using a rotary evaporator (BÜCHI Labortechnik AG, Flawil, Switzerland). The concentrated extracts were then freeze-dried using a freeze-drier (Labconco Corporation, Kansas City, MO, USA) at −52 °C and ≤0.1 MPa for 48 h. The dried extracts were ground, sealed in airtight containers, and stored at −20 °C until further analysis.

#### 2.2.2. Characterization and Biological Evaluation of MAPE Extract

##### Fractionation and Identification of Polyphenolic Compounds by HPLC

HPLC analysis was performed using an Agilent 1260 system. Separation was achieved on a Zorbax Eclipse Plus C8 column (4.6 × 250 mm, 5 µm). The mobile phase consisted of water (A) and 0.05% trifluoroacetic acid in acetonitrile (B), delivered at a flow rate of 0.9 mL/min. A linear gradient was applied as follows: 0–1 min (82% A), 1–11 min (75% A), 11–18 min (60% A), and 18–24 min (82% A). Detection was performed at 280 nm, with an injection volume of 5 µL, and the column temperature was maintained at 40 °C. Phenolic compounds were identified using authentic analytical reference standards. A mixed standard solution containing the investigated phenolic compounds was analyzed under the same chromatographic conditions as the samples. Compound identities were assigned by comparing the retention times of the chromatographic peaks in the samples with those of the corresponding reference standards.

##### Determination of Antioxidant Activity

The DPPH^•^ radical scavenging activity was evaluated according to the method described by Mahmoud et al. [29]. Briefly, 4.9 mL of DPPH solution (6.5 × 10^−5^ mol L^−1^) prepared in methanol was mixed with 100 μL of MAPE solution (1 mg/mL), vortexed thoroughly, and incubated in the dark at room temperature for 30 min. Methanol (100 μL) served as the control. After incubation, the absorbance at 517 nm was measured using a UV–Vis spectrophotometer (Cary 100, Agilent Technologies, Santa Clara, CA, USA). The free radical scavenging activity (%) was calculated using the following equation:

Scavenging activity (%) = (1 − *A_sample_*/*A*_0_) × 100 (1)

where *A_sample_* and *A*_0_ are the absorbance measurements at 517 nm for the sample and control, respectively.

The ABTS^•+^ radical scavenging activity was determined according to the method described by Re et al. [30]. Briefly, 300 μL of MAPE solution (1 mg/mL) was mixed thoroughly with 4.7 mL of ABTS solution (adjusted to an absorbance of 0.70 ± 0.02 at 734 nm), vortexed, and incubated in the dark at room temperature for 30 min. Anhydrous ethanol (300 μL) was used as the control. After incubation, the absorbance was measured at 734 nm using a UV–Vis spectrophotometer (Cary 100, Agilent Technologies, Santa Clara, CA, USA). The free radical scavenging activity (%) was calculated using the following equation:

Scavenging activity (%) = (1 − *A_sample_*/*A*_0_) × 100 (2)

where *A_sample_* and *A*_0_ are the absorbance measurements at 734 nm for the sample and control, respectively.

##### Antimicrobial Activity Evaluation

The antimicrobial activity of MAPE was evaluated using the broth dilution method according Osés et al. [31]. A volume of 100 µL of microbial suspension (10^6^ CFU/mL) was inoculated into 10 mL of nutrient broth (for bacteria) or potato dextrose broth (for fungi). MAPE was added to achieve a final concentration of 50 µg/mL. Negative controls (medium containing only microorganisms) and positive controls (ampicillin for bacteria and fluconazole for fungi) were included. After incubation for 24 h at optimal temperatures, microbial growth was determined by measuring absorbance at 600 nm. The following equation calculates inhibition (%):

Inhibition (%) = 100 − [(OD sample/OD negative control) × 100](3)

where OD sample is the optical density of the microbial culture treated with MAPE, and OD negative control is the optical density of the untreated microbial culture (negative control).

Also, the minimum inhibitory concentration (MIC) values were determined using the same procedure with different concentrations of MAPE (25–200 µg/mL) to identify the lowest concentration that inhibited visible microbial growth.

##### Antiviral Activity Evaluation of MAPE

HAV was kindly provided by the Faculty of Medicine, Al-Azhar University, Girls Branch. The virus was propagated in Vero cells (ATCC CCL-81) with 5% CO_2_ at 37 °C with daily examination until cytopathic effect (CPE) appears. Then, the virus was released from infected cells by three successive rounds of freezing and thawing, followed by centrifugation at 5000× *g* at 4 °C for 5 min to remove cells debris. The supernatant was separated, and the stock solution of HAV was stored at −80 °C until needed. The titer of HAV was determined by plaque assay according to Cromeans et al. [32]. Briefly, Vero cells were cultured in six-well cell culture plates seeded with 5 × 105 cells/mL. The cells were infected using ten-fold serial dilutions of the HAV viral stocks and incubated with 5% CO_2_ at 37 °C for 90 min. The semisolid overlay made by combining equal volume of 2× MEM-E medium (Biowest, Nuaillé, France) with 2× agarose (Biobasic, Markham, ON, Canda), to achieve a final concentration of 1×, was added to the cells after the inoculum had been removed after 90 min of incubation. Each well was filled with 3 mL of the semisolid mixture. Next, the plates were incubated with 5% CO_2_ at 37 °C for 8 days. At the end of incubation period, the agarose overlay was removed, and 3 mL of crystal violet staining solution (1% crystal violet and 20% methanol in distilled water) was applied for 30–60 s to stain the cells after they had been fixed with 10% formaldehyde. Plaque-forming units (PFUs) were then computed once the plaques had been counted to determine the viral titer (PFU/mL).

The cytotoxicity of MAPE was evaluated using a 3-(4,5-dimethylthiazol-2-yl)-2,5-diphenyltetrazolium bromide (MTT) assay. Vero cells (ATCC CCL-81) were cultured at 37 °C in a humidified 5% CO_2_ atmosphere until a confluent monolayer was obtained. Cells were seeded at a density of 5 × 10^4^ cells/well in 96-well plates (Bio Basic, Markham, ON, Canada) and incubated for 24 h. Subsequently, cells were treated with serial dilutions of the extract (0.1 mL of each tested dilution/well) and incubated for 48–72 h. After incubation, the medium was removed by aspiration, and 20 µL of MTT solution (5 mg/mL) (Bio Basic, Canada) was added to each well. The plates were shaken at 150 rpm for 5 min and incubated for 4 h at 37 °C. The MTT solution was then discarded, and 200 µL of dimethyl sulfoxide (DMSO) was added to dissolve the formazan crystals. The plates were shaken for 5 min and incubated for an additional 30 min. Absorbance was measured at 560 nm with background subtraction at 620 nm using a microplate reader. The following equation calculates cytotoxicity (%):

Cytotoxicity = (OD control − OD treated)/(OD control) × 100(4)

Moreover, the antiviral activity (IC_50_) of MAPE was determined according to Boivin et al. [33]. Vero cells were seeded at 5 × 10^4^ cells/mL in 96-well plates and incubated overnight. Equal volumes (1:1, *v*/*v*) of non-toxic concentrations of the extract and the hepatitis A virus (HAV) suspension were co-incubated at 37 °C for 1 h. Then, 100 µL of the virus/extract mixture was added to each well and incubated at 37 °C in a 5% CO_2_ atmosphere for up to 8 days. Virus-infected untreated cells and uninfected untreated cells were used as the virus and cell controls, respectively. Following the incubation period, cell viability was evaluated using the MTT assay, as previously described. Absorbance was recorded at 560 nm with background correction at 620 nm. The half-maximal inhibitory concentration (IC_50_) was determined by comparing the viability of treated cells with that of the corresponding control groups, as follows:

Viral Inhibition Rate = ((ODtv − ODcv))⁄((ODcd − ODcv) × 100)(5)

where ODtv is the absorbance of virus-infected cells treated with the test extract, ODcv is the absorbance of the untreated virus control, and ODcd is the absorbance of the untreated cell control.

##### Molecular Docking

Molecular docking simulations were performed to study the binding interactions of the identified phenolic compounds with the target enzyme using the Molecular Operating Environment (MOE 2015) program (Chemical Computing Group Inc., Montreal, QC, Canada). The three-dimensional structure of hepatitis A virus (HAV) 3C protease (PDB ID: 2CXV; “https://www.rcsb.org/ (accessed on 19 May 2026)” is available from the RCSB Protein Data Bank. The MOE ‘QuickPrep’ module was utilized to optimize the protein architecture prior to docking. Hydrogen atoms were added, and the protonation states of ionizable residues (Lys, Arg, Asp, Glu, His) were assigned using the Protonate 3D function at pH 7.0 to simulate physiological conditions. To lessen the influence of steric interactions and improve the structure of hydrogen bonds, energy reduction was performed using the AMBER10: EHT force field. Water molecules observed in crystallography were not included, as their structure was proven to be unconnected with the design of the binding site in this system. No cofactors, ions, metal atoms, or missing residues or atoms were present in the structure; hence, no reconstruction was necessary. The chemical structures of the compounds under consideration were initially drawn in ChemDraw 18.0 and then saved in MDL molfile format for docking investigations. The binding affinities, interacting amino acids, hydrogen bond lengths, and types of interactions were analyzed to gain insight into the binding mechanisms of proteins with substances.

#### 2.2.3. Preparation of SAlg-WP Hydrogel Beads Encapsulating Freeze-Dried MAPE

**Figure 1:**
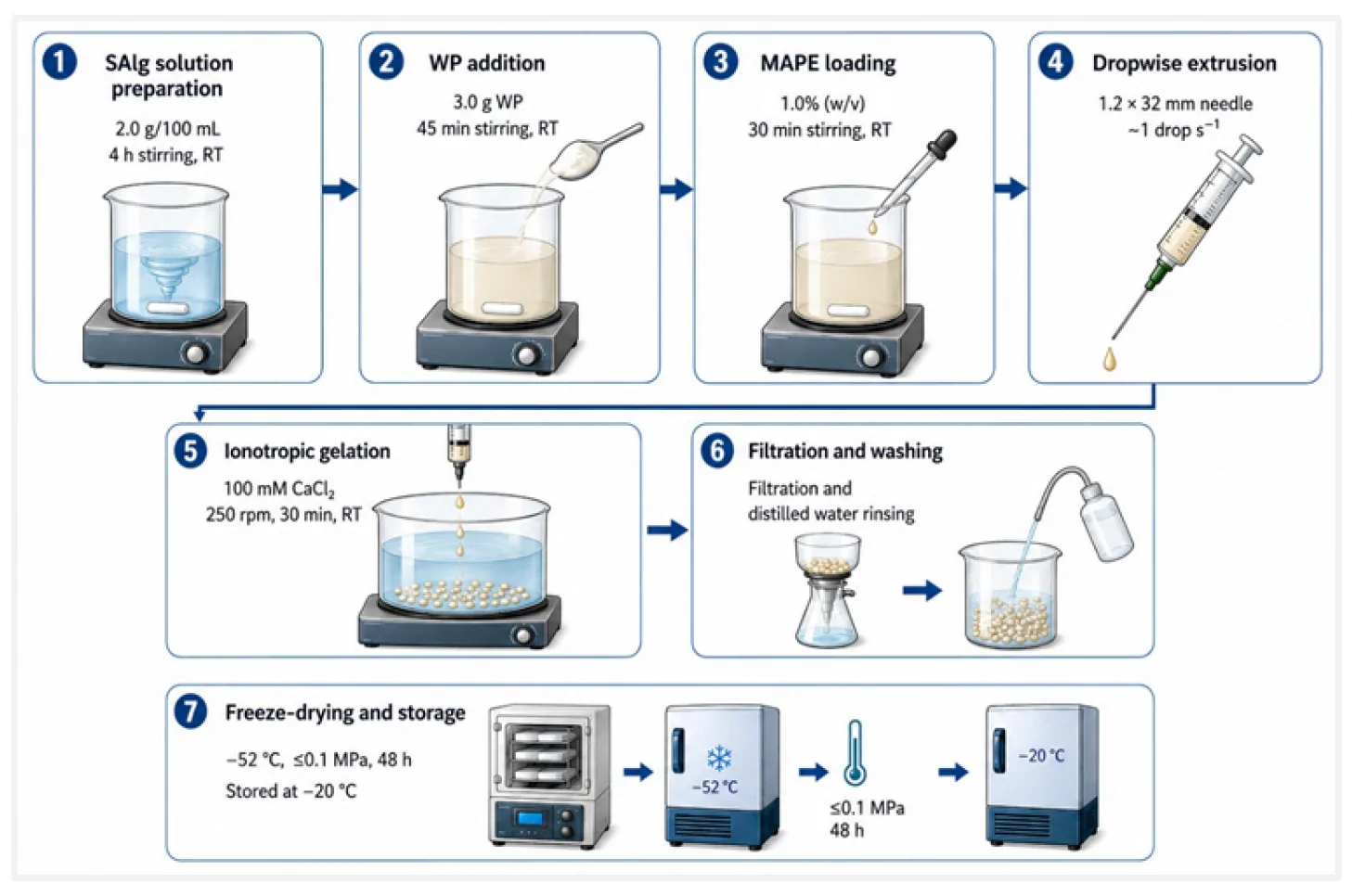
Schematic illustration of the preparation of SAlg/WP-MAPE hydrogel beads. SAlg, sodium alginate; WP, whey protein concentrate; MAPE, *M. argun* phenolic extract.

Hydrogel beads were prepared using a modified ionotropic gelation method with calcium chloride (CaCl_2_) as the cross-linking agent, following established procedures for alginate-based systems [25]. Sodium alginate (SAlg, 2.0 g) was dissolved in 100 mL of distilled water under continuous magnetic stirring at room temperature (RT) for 4 h until a homogeneous solution was obtained. Whey protein concentrate (WP, 3.0 g) was gradually added to the SAlg solution and stirred for an additional 45 min at RT to ensure complete dispersion. MAPE was incorporated into the SAlg/WP mixture at 1.0% (*w*/*v*), and the mixture was stirred continuously for 30 min to obtain a uniform hydrated suspension. The resulting SAlg/WP-MAPE mixture was transferred into a syringe fitted with a stainless steel needle (1.2 × 32 mm) and extruded dropwise at a rate of approximately 1 drop s^−1^. The droplets were released from a height of 7 cm into a 100 mM CaCl_2_ solution, maintained under gentle magnetic stirring (250 rpm) at RT, to enable bead formation via ionic cross-linking. After extrusion, the hydrogel beads were kept in the CaCl_2_ solution for an additional 30 min to ensure complete gelation. The beads were then collected by filtration and thoroughly rinsed with distilled water to remove residual CaCl_2_. A portion of the freshly prepared beads was freeze-dried at −52 °C under ≤0.1 MPa for 48 h and subsequently stored at −20 °C until further analyses. Figure 1 shows a schematic of the hydrogel bead preparation process.

##### Encapsulation Efficiency Determination

To determine the efficiency of encapsulation according to Feng et al. [25], 10 mg of the beads was dispersed in 5 mL of 5% (*w*/*v*) sodium citrate solution and sonicated for 30 min. The resulting suspension was then centrifuged at 3000 rpm for 10 min. The encapsulation efficiency of MAPE in sodium alginate–whey protein (SAlg/WP) beads was quantified using the Folin–Ciocalteu assay and calculated according to the following equation:

Encapsulation Efficiency (%) = (TPC beads)/(TPC extract) × 100(6)

where TPC beads represent the total content of phenols encapsulated in beads, and TPC extract represents the total content of phenols of the extract.

##### Fourier Transform Infrared Spectroscopy (FTIR)

FTIR spectra were recorded using an ATR-FTIR spectrometer (Thermo Fisher Scientific, Waltham, MA, USA) fitted with a diamond crystal. Dried microcapsule samples were directly placed on the ATR crystal, and spectra were collected over 4000–400 cm^−1^ at a resolution of 4 cm^−1^ with 32 accumulated scans. A background spectrum was obtained before each analysis and automatically subtracted from the sample spectrum. Spectral features, including band positions and characteristic peaks, were examined to identify potential intermolecular interactions following established procedures.

##### Scanning Electron Microscopy (SEM)

The surface and structural morphology of the prepared formulations was examined using high-resolution SEM. Analysis was performed using an FEI Quanta FEG 250 instruments (Thermo Fisher Scientific, Waltham, MA, USA). For sample preparation, approximately 1 g of each freeze-dried formulation was mounted onto aluminum stubs using double-sided adhesive tape and subsequently coated with a thin layer of gold using a sputter coater to enhance conductivity. Micrographs were obtained at appropriate accelerating voltages.

#### 2.2.4. Preparation and Characterization of Fortified Yoghurt

Fortified yoghurt was prepared according to the method described by Kurbonova et al. [34], with slight modifications. Low-fat buffalo milk (13.2% total solids, 4.1% protein, 0.5% fat, and 4.7% lactose) was pasteurized at 80 °C for 20 min and subsequently cooled to 42 °C. The milk was inoculated with 2% (*w*/*v*) yoghurt starter culture consisting of *Streptococcus thermophilus* and *Lactobacillus delbrueckii* subsp. *Bulgaricus* and mixed thoroughly. SAlg/WP-MAPE was then incorporated into the inoculated milk at 5%, 10%, or 15% (*w*/*w*) to obtain fortified yoghurt samples designated Y-5, Y-10, and Y-15, respectively. A control yoghurt without SAlg/WP-MAPE was prepared under identical processing conditions. The mixtures were dispensed into sterile sealed glass jars and incubated at 42 °C until the pH reached 4.60 (3 h). After fermentation, the yoghurt samples were immediately cooled and stored at 4 ± 1 °C for 1, 7, and 14 days.

##### Color Analysis

Color measurements were performed using a Color Quest XE colorimeter (HunterLab, Reston, VA, USA). The color attributes of yoghurt samples with and without sodium alginate–whey protein (SAlg/WP)-encapsulated MAPE were evaluated by determining the CIELAB color parameters L* (lightness), a* (redness/greenness), and b* (yellowness/blueness). Chroma (C*) and the total color difference (ΔE) were subsequently calculated using the following equations:

(7) $$C^{*}=\sqrt{a^{*2}+b^{*2}}$$

(8) $$∆E=\sqrt{\Delta {\left(L^{*}\right)}^{2}+\Delta {\left(a^{*}\right)}^{2}+\Delta {\left(b^{*}\right)}^{2}}$$

##### pH and Syneresis Determinations

The pH values of yoghurt samples stored at 4 °C for 1, 7, and 14 days were determined using a pH meter (HANNA pH 20, Hanna Instruments, Woonsocket, RI, USA) calibrated at 25 °C.

Syneresis of the yoghurt samples was determined by centrifugation according to a previously reported method, with slight modifications [35]. Briefly, 10 g of yoghurt was accurately weighed into a centrifuge tube and centrifuged at 4000 rpm for 20 min at room temperature (Shanghai Lu Xiangyi Centrifuge Instrument Co., Shanghai, China). After centrifugation, the separated whey (supernatant) was carefully decanted by inverting the centrifuge tube, and its weight was recorded. The syneresis index was expressed as the percentage of whey released relative to the initial weight of the yoghurt sample and calculated using the following equation:

(9) Synersis%=m2/m1×100

where m1 and m2 are the mass of the yoghurt sample and the supernatant, respectively.

##### Preparation of Water-Soluble Extract from Yoghurt Samples for Antioxidant Determinations

To evaluate the antioxidant capacity of the yoghurt samples, the water-soluble extracts were first prepared [36]. For each yoghurt sample, the pH was adjusted to 4.6 using either 1.0 M NaOH or 1.0 M HCl. The samples were then centrifuged at 11,739× *g* for 15 min at room temperature. After centrifugation, the supernatant was collected and centrifuged again under the same conditions to improve clarity. The clarified supernatants were then filtered through a 0.45 μm membrane filter. The final filtrate was used for measuring antioxidant activity, as described under the determination of antioxidant activity section.

#### 2.2.5. Statistical Analysis

All experiments were performed in triplicate, and the results are expressed as mean ± standard deviation (SD). Statistical analyses were carried out using SPSS software (Version 20.0, IBM Corp., Armonk, NY, USA). Depending on the experimental design, differences among treatments were evaluated using one-way or two-way analysis of variance (ANOVA). When significant differences were observed, Duncan’s multiple range test was used for post hoc multiple comparisons. Differences were considered statistically significant at *p* < 0.05.

## 3. Results and Discussion

### 3.1. HPLC Analysis of Phenolic Compounds

**Figure 2:**
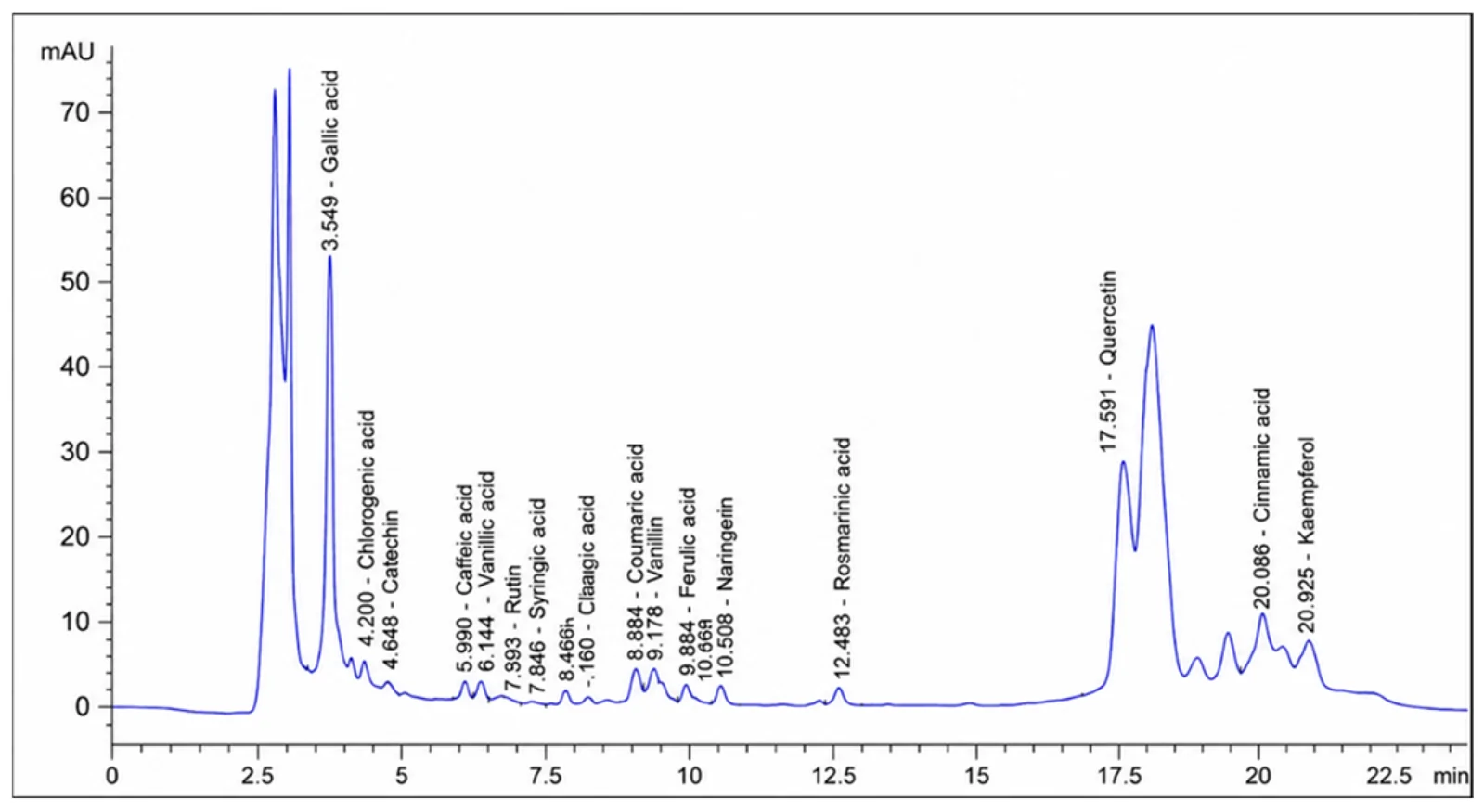
HPLC chromatogram of phenolic compounds identified in *M. argun* phenolic extract (MAPE).

The phenolic profile of MAPE as determined by HPLC analysis is shown in Figure 2. The extract was characterized by the presence of several phenolic acids and flavonoids, with considerable variation in their abundance. Among the identified phenolic acids, gallic acid was the predominant compound (651.08 µg/g), followed by cinnamic acid and rosmarinic acid. In contrast, moderate amounts of p-coumaric acid, ferulic acid, and chlorogenic acid were also detected. Lower concentrations of caffeic acid, syringic acid, vanillin, and ellagic acid were identified. Similar phenolic profiles rich in gallic acid and hydroxycinnamic acid derivatives have been reported in palm species and other desert-adapted plants, where these metabolites may contribute to oxidative stress tolerance and protective responses under harsh environmental conditions [37].

Flavonoid analysis revealed that quercetin was the dominant flavonoid compound (2833.01 µg/g), followed by kaempferol, whereas rutin, catechin, and naringenin were detected at lower levels. The predominance of quercetin is particularly important because flavonoids with multiple hydroxyl groups and conjugated aromatic structures exhibit strong radical-stabilizing and electron transfer properties, which may significantly contribute to the biological activity of the extract [38]. In addition to its antioxidant potential, quercetin has been extensively reported for its antimicrobial, anti-inflammatory, and antiviral activities, supporting the multifunctional properties of MAPE.

The detected phenolic composition suggests that the biological activity of MAPE is likely associated with synergistic interactions among phenolic acids and flavonoids rather than the action of a single compound alone. Previous studies have demonstrated that complex phenolic mixtures often exhibit enhanced antioxidant and antimicrobial activity due to additive and synergistic effects among flavonoid and phenolic acid constituents [3]. Furthermore, the presence of chlorogenic acid, rosmarinic acid, rutin, and ferulic acid may contribute to the extract’s antiviral potential, as these compounds have previously been reported to interfere with viral replication pathways and viral enzyme activity. Overall, the predominance of flavonoids and hydroxycinnamic acid derivatives indicates that *M. argun* represents a promising natural source of multifunctional bioactive compounds suitable for encapsulation and functional food applications.

The antioxidant activity of MAPE was evaluated using DPPH^•^ and ABTS^•+^ radical-scavenging assays, which showed strong scavenging activities of 82.62% and 57.38%, respectively. The higher scavenging activity observed in the DPPH assay than in the ABTS assay may be attributed to differences in radical chemistry, reaction mechanisms, and solvent compatibility. DPPH radicals are predominantly scavenged via hydrogen atom transfer in organic media, whereas ABTS radicals can react with both hydrophilic and lipophilic antioxidants in both aqueous and organic systems [3]. Consequently, variations between DPPH and ABTS scavenging activities are common in phenol-rich plant extracts and reflect the complexity of their antioxidant constituents rather than differences in antioxidant effectiveness [29].

The pronounced antioxidant activity of MAPE is closely associated with its phenolic profile, as revealed by HPLC analysis, particularly the high abundance of quercetin and gallic acid. These compounds possess multiple hydroxyl groups and conjugate aromatic structures that facilitate hydrogen atom donation, electron transfer, and stabilization of free radicals, thereby contributing substantially to antioxidant activity [39]. Furthermore, other identified phenolics, including chlorogenic acid, ferulic acid, rosmarinic acid, catechin, and kaempferol, are recognized as potent antioxidants and may collectively enhance the extract’s radical-scavenging capacity through complementary and synergistic interactions among flavonoids and phenolic acids.

The antioxidant activity observed in the present study is consistent with previous reports on *M. argun* fruits. The extracts from different parts of *M. argun* exhibited considerable antioxidant activity, attributed to their high phenol and flavonoid content. Although direct quantitative comparisons are limited by differences in plant material, extraction solvents, extraction procedures, and antioxidant assays, the present findings further confirm that *M. argun* is a rich source of naturally occurring antioxidant compounds. Moreover, although previous researchers investigated fruit-derived polysaccharides rather than phenolic extracts, they likewise reported antioxidant activity associated with *M. argun*-derived biopolymers, further supporting the overall antioxidant potential of this underexplored desert palm [8]. In addition, comparative evaluation [7] indicated that *M. argun* exhibited antioxidant activity comparable to that of *Hyphaene thebaica* (doum), highlighting its potential as an alternative palm-derived source of natural antioxidants for functional food applications.

Recent evidence suggests that the antioxidant capacity of plant extracts depends on the collective contribution of multiple phenolic constituents rather than on a single predominant compound [39]. Accordingly, the high antioxidant activity of MAPE is likely attributable to synergistic interactions among its diverse phenolic compounds, which enhance the extract’s overall radical-scavenging capacity. These findings, together with the rich phenolic profile identified in *M. argun*, underscore its potential as a multifunctional natural antioxidant for encapsulation and incorporation into functional food systems.

### 3.2. Antimicrobial Activity

Testing the antimicrobial activity of MAPE against Gram-positive bacteria (*Bacillus cereus*, *Staphylococcus aureus*, and *Bacillus subtilis*), Gram-negative bacteria (*Escherichia coli* and *Pseudomonas aeruginosa*), and fungal strains (*Candida albicans* and *Aspergillus niger*) showed that the extract possessed broad-spectrum inhibitory activity against all tested microorganisms (Table 1 and Table 2). However, microbial susceptibility varied according to microorganism type and extract concentration. Among the tested strains, *Bacillus subtilis* and *Candida albicans* exhibited the highest sensitivity toward MAPE, whereas *E. coli* showed comparatively lower susceptibility.

In general, Gram-positive bacteria were more susceptible to MAPE than Gram-negative bacteria. This difference may be associated with structural variations in the bacterial cell envelope, as Gram-negative bacteria possess an outer lipopolysaccharide membrane that restricts the penetration of hydrophobic phenolic compounds and limits intracellular accumulation of bioactive compounds. In contrast, the relatively porous peptidoglycan layer of Gram-positive bacteria facilitates interactions between phenolic compounds and cellular targets, thereby increasing antimicrobial sensitivity [40,41].

The antimicrobial activity of MAPE is likely due to the synergistic action of its major phenolic constituents, particularly quercetin, gallic acid, ferulic acid, chlorogenic acid, catechin, and kaempferol, as identified by HPLC analysis. These compounds contain multiple hydroxyl groups and hydrophobic aromatic structures that can interact with microbial cell membranes, altering membrane permeability, disrupting enzyme activity, and inducing oxidative damage within microbial cells. Previous studies demonstrated that flavonoids, such as quercetin and catechin, can destabilize membrane integrity and interfere with nucleic acid synthesis, whereas phenolic acids, including gallic acid and ferulic acid, may inhibit essential metabolic pathways and energy production [42].

The inhibitory activity of MAPE increased progressively with increasing concentration up to 100 µg/mL, suggesting enhanced interaction between phenolic compounds and microbial cells at moderate concentrations. However, a slight reduction in antimicrobial efficiency was observed at concentrations above 100 µg/mL. This behavior may be associated with aggregation phenomena or reduced diffusion efficiency of phenolic compounds at higher concentrations, which could limit their accessibility to microbial targets [43].

Regarding antifungal activity, *Candida albicans* exhibited greater susceptibility than *A. niger*. The lower sensitivity of *A. niger* may be associated with the complex filamentous structure and spore-forming characteristics of Aspergillus species, which confer additional resistance to external stressors and antimicrobial agents. In contrast, the relatively simpler cellular organization of Candida may facilitate penetration and interaction of phenolic compounds with fungal cell membranes, particularly through interactions with ergosterol-containing membrane structures.

Overall, the results indicate that MAPE exhibits considerable antimicrobial activity, attributable to its multifunctional phenolic composition. In addition to their antioxidant and antiviral properties, these phenolic compounds improve microbiological stability and functional performance in fortified food systems, supporting the potential application of MAPE as a natural, multifunctional ingredient in functional dairy products.

### 3.3. Antiviral Activity

**Figure 3:**
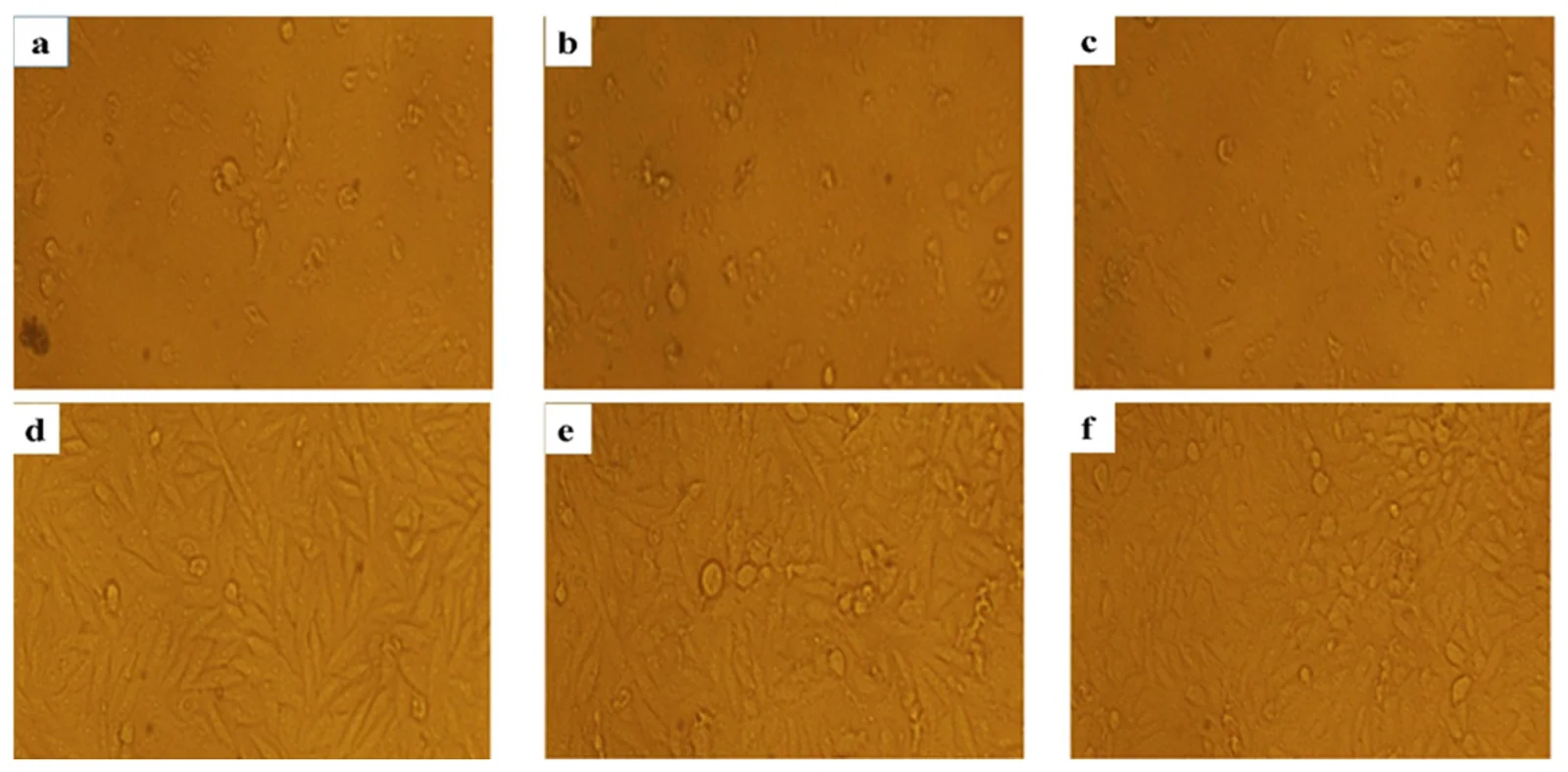
Effect of different concentrations of MAPE on the viability and morphology of Vero cells, as observed under an inverted microscope (10× magnification). Cells were treated with serial two-fold dilutions of MAPE ranging from 1000 to 31.25 μg/mL. (**a**) 1000 μg/mL; (**b**) 500 μg/mL; (**c**) 250 μg/mL; (**d**) 125 μg/mL; (**e**) 62.5 μg/mL; (**f**) 31.25 μg/mL.

**Figure 4:**
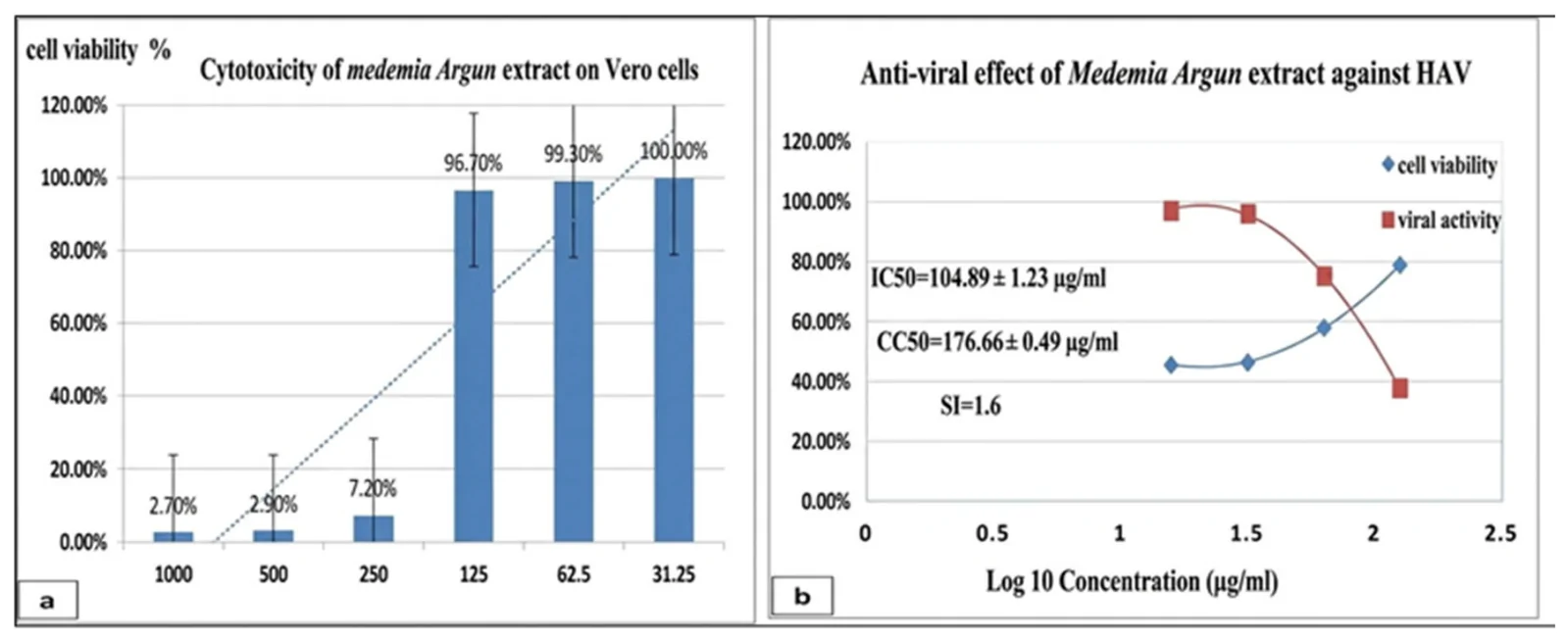
(**a**) Cytotoxic concentration (CC50) and maximum non-toxic concentration (MNTC) of MAPE for Vero cells. (**b**) Antiviral activity of MAPE against hepatitis A virus (HAV).

The cytotoxicity evaluation demonstrated that MAPE exhibited relatively low toxicity toward Vero cells, with a CC_50_ value of 176.66 ± 0.49 μg/mL and a maximum non-toxic concentration (MNTC) of 125 μg/mL, maintaining >96% cell viability (Figure 3 and Figure 4). The antiviral assay showed that MAPE exerted moderate inhibitory activity against hepatitis A virus (HAV), with an IC_50_ value of 104.89 ± 1.23 μg/mL and a selectivity index (SI) of 1.6. Although the obtained SI value indicates limited antiviral selectivity, crude plant extracts often exhibit moderate selectivity due to complex mixtures containing both active and inactive phytochemicals [44]. Therefore, further purification and fractionation of the extract may improve antiviral efficacy while reducing cytotoxicity.

The observed antiviral activity may be associated with the phenolic composition of MAPE, particularly quercetin, rutin, chlorogenic acid, rosmarinic acid, gallic acid, and kaempferol, as identified by HPLC analysis. These phenolic compounds have previously been reported to interfere with viral replication by inhibiting viral enzymes, suppressing viral protein synthesis, and modulating oxidative stress pathways associated with RNA viral infections [45,46].

### 3.4. Molecular Docking Analysis with HAV 3C Protease (PDB ID: 2CXV)

**Figure 5:**
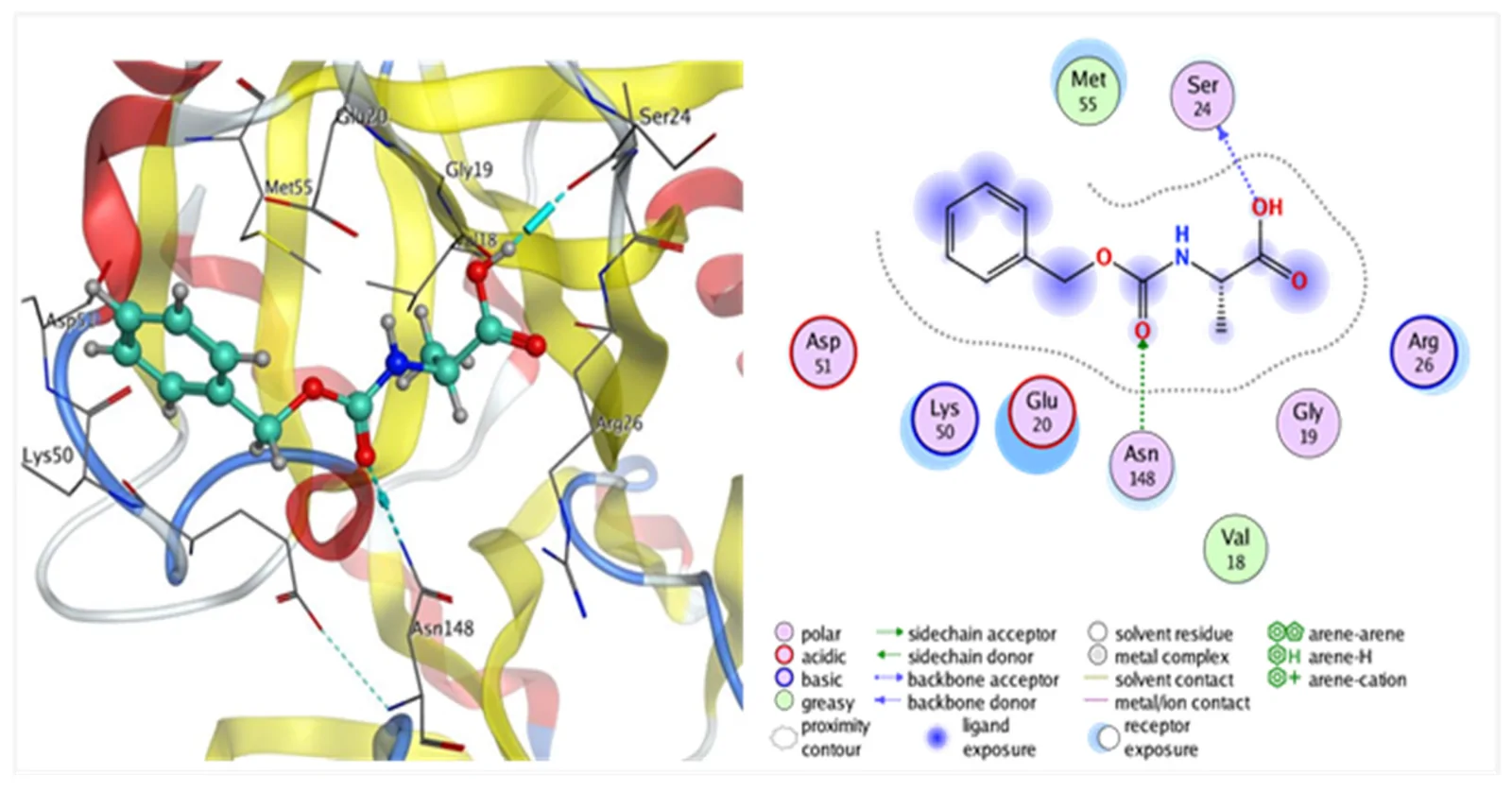
Three-dimensional (3D) and two-dimensional (2D) interaction of the reference ligand (BBL) with the active site of 2CXV.

**Figure 6:**
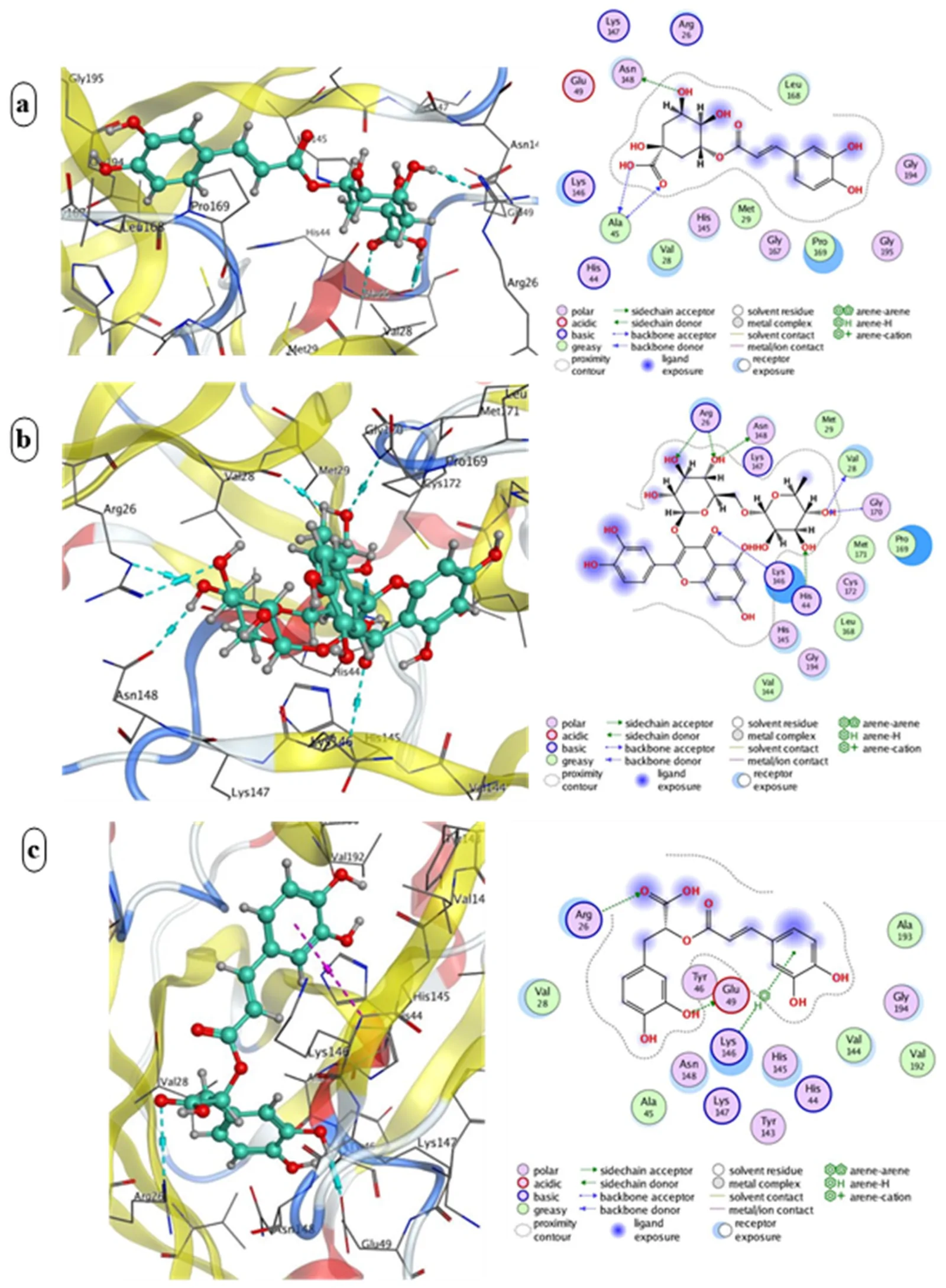
Three-dimensional (3D) and two-dimensional (2D) molecular interactions of (**a**) chlorogenic acid, (**b**) rutin, and (**c**) rosmarinic acid with the active site of 2CXV.

**Figure 7:**
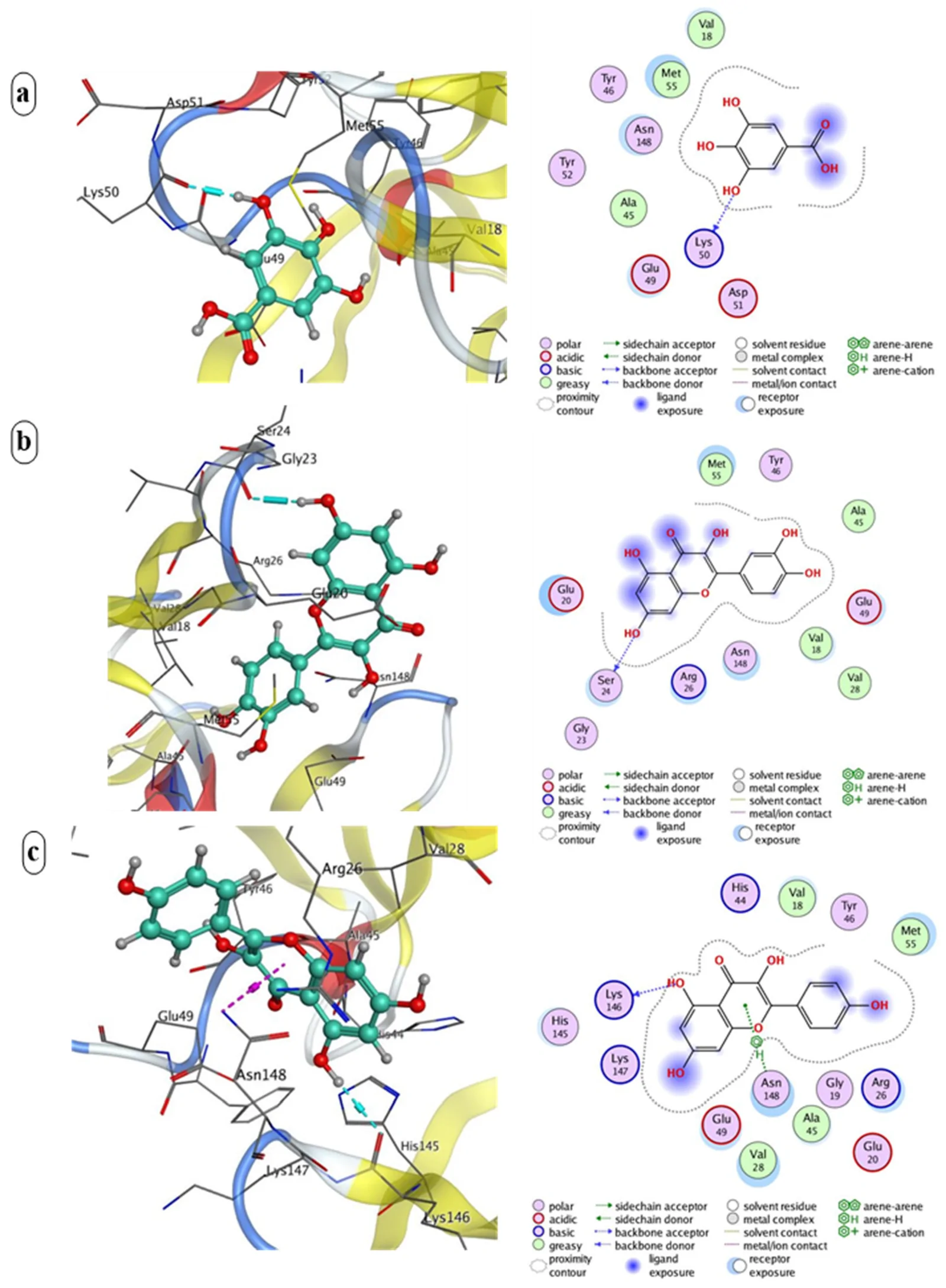
Three-dimensional (3D) and two-dimensional (2D) molecular interactions of (**a**) gallic acid, (**b**) quercetin, and (**c**) kaempferol with the active site of 2CXV.

Molecular docking simulations were conducted using MOE 2015 software to investigate the binding interactions between the compounds investigated (1–18) and the target enzyme. Key parameters including binding affinities, interacting amino acid residues, hydrogen bond lengths, and interaction types were analyzed to elucidate the compound–protein binding mechanisms. The three-dimensional crystal structure of the target protein was retrieved from the RCSB Protein Data Bank “https://www.rcsb.org/ (accessed on 23 February 2026)”. Prior to docking, the protein was prepared by removing water molecules and any co-crystallized ligands. The chemical structures of compounds (**1**–**18**) were generated using ChemDraw 18.0 and subsequently saved in MDL molfile format for docking studies. The binding energies, interacting amino acid residues, bond strengths, and bond lengths for the complexes formed between compounds (**1**–**18**) and the HAV 3C protease (2CXV) active site are presented in Table 3. To validate the reliability of the docking protocol, the co-crystallized ligand N-[(benzyloxy)carbonyl]-L-alanine (BBL) was re-docked into the enzyme’s binding pocket. The native ligand exhibited a binding affinity of −4.74 kcal/mol and formed two hydrogen bonding interactions: an H-donor interaction with Ser 24 and an H-acceptor interaction with Asn 148 (Figure 5), as detailed in Table 3. The tested compounds (**1**–**18**) demonstrated a wide range of binding affinities, with docking scores spanning from −4.21 to −7.29 kcal/mol. This variability reflects differing degrees of complementarity with the HAV 3C protease (2CXV) active sites. The binding patterns predominantly involved hydrogen bond formation with key amino acid residues, which are essential for stabilizing the ligand–enzyme complexes. Among all the evaluated compounds, rutin (compound **7**) exhibited the most favorable binding energy, scoring −7.29 kcal/mol, which was substantially superior to that of the native ligand (BBL) (−4.74 kcal/mol). Remarkably, rutin established an extensive interaction network comprising seven hydrogen bonds with multiple amino acid residues. These interactions included an H-donor bond with Val 28 and H-acceptor bonds with Asn 148, Gly 170, and Arg 26 (Figure 6). Chlorogenic acid (compound **2**) demonstrated a strong binding affinity of −6.37 kcal/mol, forming three hydrogen bonds with Ala 45 and Asn 148. The interaction involved H-donor bonds (Figure 6). Rosmarinic acid (compound **13**) also showed notable binding energy of −6.04 kcal/mol, engaging in multiple interactions including an H-donor bond with Glu 49, an H-acceptor bond with Arg 26, and a pi-H interaction with Lys 146 (Figure 6). The extracted compounds with the highest percentages in the extract demonstrated moderate binding affinities in the range of −4.34 to −4.89 kcal/mol. Quercetin (compound **15**) scored −4.89 kcal/mol with an H-donor bond to Ser 24 (Figure 7). Gallic acid (compound **1**) displayed a binding energy of −4.34 kcal/mol, forming a single H-donor interaction with Lys 50 (Figure 7). Kaempferol (compound **17**) scored −4.35 kcal/mol, binding to Lys 146 and Asn 148 through H-donor and pi-H interactions (Figure 7). In the docking analysis, ellagic acid (compound **8**) and catechin (compound **3**) exhibited binding affinities of −5.33 kcal/mol and −5.17 kcal/mol, respectively. Ellagic acid formed two hydrogen bonds with Asn 148 and His 44, supplemented by a pi-H interaction with Lys 146. Catechin established a single H-donor interaction with Val 144. Naringenin (compound **12**) displayed a binding energy of −5.02 kcal/mol, forming a hydrogen bond with Lys 146. Ferulic acid (compound **11**) exhibited a binding affinity of −4.75 kcal/mol through an H-acceptor interaction with His 145. Coumaric acid (compound **9**) showed −4.58 kcal/mol, engaging in an H-donor bond with Ala 45 and a pi-H interaction with Gly 19. Vanillin (compound **10**) demonstrated a binding affinity of −4.36 kcal/mol, forming H-donor and H-acceptor bonds with Lys 146 and Arg 26, respectively. Caffeic acid (compound **5**) achieved −4.43 kcal/mol, forming three H-donor bonds with Val 28 and Val 144. Syringic acid (compound **6**) and cinnamic acid (compound **16**) recorded the lowest binding affinities at −4.29 and −4.21 kcal/mol, respectively, with interactions involving Lys 146, Arg 26, and Asn 148.

The current docking study looked at 18 phenolic compounds to see if they could inhibit HAV 3C protease (2CXV). The results showed that rutin (7), chlorogenic acid (2), and rosmarinic acid (13) had the greatest binding affinities (−7.29, −6.37, and −6.04 kcal/mol, respectively). They formed stable hydrogen bond networks with important active site residues. There is much evidence from past studies that these chemicals can fight viruses by targeting different viral proteins. Rutin (7), which exhibited the best performance in this study, has been found to be a powerful inhibitor of SARS-CoV-2 RNA-dependent RNA polymerase (RdRp), with an IC_50_ of 60.09 nM, compared to remdesivir in in vitro studies [47]. Essaadi et al. [48] also showed that rutin binds well to rhinovirus 3C protease (−8.0 kcal/mol), engaging all three catalytic residues. This is quite similar to what we found with HAV 3C protease. Previous computer studies have demonstrated that rosmarinic acid (13) is antiviral against a wide range of viruses. Sujitha and Murugesan [49] found docking scores as low as −10.0 kcal/mol with dengue virus proteins. El-Sayed et al. [50] found it to be the best inhibitor of the SARS-CoV-2 major protease. Tsilimingkra and Papaneophytou [51] experimentally confirmed rosmarinic acid by showing that it inhibited rhinovirus 3C protease activity by more than 55% in vitro. This directly supports our docking results. Chlorogenic acid (2) has been previously acknowledged for its antiviral properties. El-Sayed et al. [50] characterized it as a high-affinity ligand for SARS-CoV-2 Mpro and RBD, whereas Jiang et al. [52] documented its synergistic antiviral activity when used in conjunction with other compounds. Quercetin (15) and kaempferol (17), although they showed low binding affinities in this study (−4.89 and −4.35 kcal/mol), have exhibited notable antiviral properties in other investigations. Parvez et al. [53] indicated that quercetin and kaempferol achieved up to 70% suppression of HBV antigen synthesis, equivalent to lamivudine. Yi et al. [54] demonstrated that quercetin directly binds to the SARS-CoV-2 S-protein domain that binds to the ACE2 receptor, indicating both virus-neutralizing and receptor-blocking processes. It is important to note that the highest predicted binding affinity does not necessarily correspond to the most abundant phenolic constituent of MAPE. While rutin exhibited the strongest predicted binding to HAV 3C protease, quercetin and gallic acid were among the most abundant compounds identified by HPLC. This difference may reflect the fact that the contribution of an individual phenolic compound to the biological activity of a crude extract depends not only on its affinity toward a specific molecular target, but also on its abundance and availability within the extract. Moreover, the combined presence of multiple phenolic compounds may result in additive or synergistic interactions, with different constituents potentially contributing through complementary mechanisms. Therefore, the docking results should be considered supportive mechanistic evidence rather than a direct predictor of the antiviral activity of the whole MAPE extract.

### 3.5. Encapsulation Efficiency

High encapsulation efficiency (EE%) of MAPE within the sodium alginate–whey protein (SAlg/WP) delivery system is essential for minimizing undesirable interactions between phenolic compounds and milk proteins, thereby improving the stability, retention, and controlled delivery of bioactive compounds in fortified yoghurt systems. Moreover, efficient encapsulation is particularly important for protecting phenolic compounds against degradation during yoghurt fermentation and refrigerated storage. In the present study, the encapsulation efficiency reached 78.92%, indicating the strong entrapment capability of the SAlg/WP hydrogel matrix toward *M. argun* phenols. The high encapsulation performance may be attributed to the formation of a compact three-dimensional polymeric network generated through electrostatic interactions and hydrogen bonding among sodium alginate, whey proteins, and phenolic compounds. These intermolecular interactions likely enhanced matrix cohesion and reduced phenolic diffusion from the encapsulation system, thereby improving the retention of bioactive compounds within the hydrogel structure. In addition, whey proteins may contribute to stabilization of phenolic compounds through hydrophobic interactions and non-covalent complex formation, thereby improving encapsulation stability.

Comparable findings were reported in [55], where the researchers obtained an encapsulation efficiency of 87.8% for riboflavin encapsulated within alginate–whey protein isolate microspheres. The slightly higher efficiency observed in their study may be associated with differences in core material properties, protein source (WPI versus WPC), and processing conditions, all of which can significantly influence matrix compactness and encapsulation behavior. Furthermore, encapsulation efficiencies ranging from 52% to 78% in alginate-based phenolic delivery systems, confirming the critical role of wall material composition in determining encapsulation performance [56]. Similarly, an encapsulation efficiency of 57.76% was reported for tea polyphenols encapsulated within sodium alginate–chitosan microcapsules [57,58]. The comparatively higher EE% obtained in the present study suggests that the combined SAlg/WP system provided improved retention capacity and enhanced protection of *M. argun* phenolics.

### 3.6. FTIR Analysis

**Figure 8:**
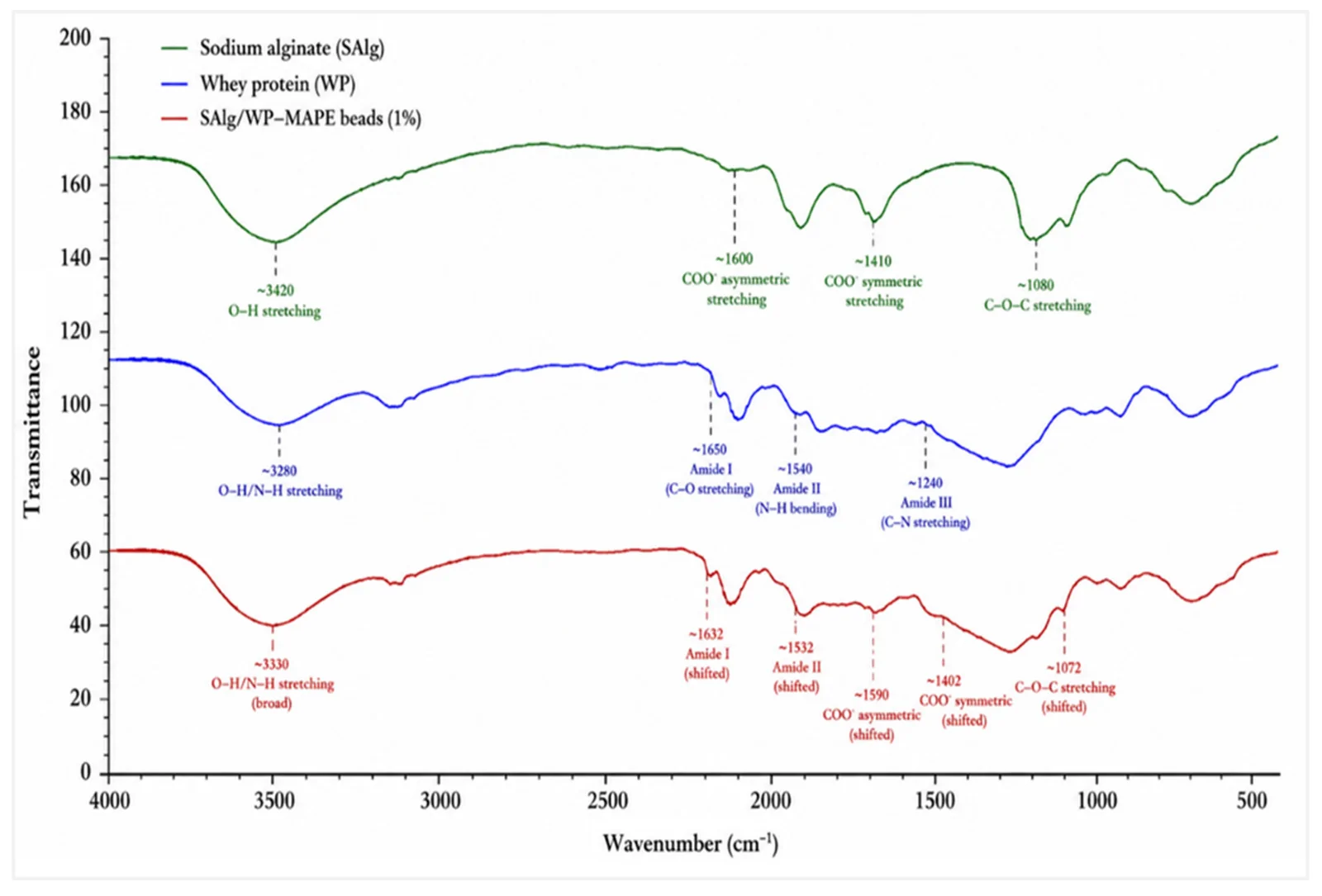
Fourier transform infrared spectroscopy (FTIR) spectra of WPC, SAlg, MAPE, and SAlg/WP–MAPE hydrogel beads.

The FTIR spectra for WP, SAlg, and SAlg/WP–MAPE beads are presented in Figure 8. WP exhibited characteristic amide I and amide II bands near 1650 and 1540 cm^−1^, respectively, corresponding to C=O stretching and N–H bending vibrations. Meanwhile, SAlg displayed typical COO^−^ asymmetric and symmetric stretching vibrations around 1600 and 1410 cm^−1^, in addition to C–O–C stretching vibrations within the 1020–1090 cm^−1^ region [59]. The FTIR spectrum of MAPE showed broad O–H stretching vibrations and aromatic-related bands characteristic of phenolic compounds [44].

Compared with the spectra of the individual components, the SAlg/WP–MAPE beads showed broadened peaks, reduced intensities, and slight shifts in the hydroxyl, amide, and carboxylate regions, indicating the formation of intermolecular interactions within the encapsulation matrix. In particular, the broad O–H absorption band within the 3200–3400 cm^−1^ region shifted toward lower wavenumbers after encapsulation, suggesting enhanced hydrogen-bonding interactions among sodium alginate, whey proteins, and phenolic compounds. Furthermore, slight shifts observed in the amide I, amide II, and COO^−^ bands indicate molecular rearrangements and electrostatic interactions within the hydrogel network.

These spectral changes provide structural evidence for the interactions of MAPE with the SAlg/WP matrix and support its incorporation within the hydrogel network. Such interactions may contribute to the retention of phenolic compounds within the polymeric matrix, consistent with the relatively high encapsulation efficiency (78.92%). Similar FTIR changes following encapsulation of phenolic compounds within alginate-based systems have previously been reported [25,57]. Thus, the FTIR results provide complementary structural information that helps explain the phenolic retention observed in the hydrogel beads and supports the interpretation of their encapsulation performance.

### 3.7. Microstructure and Surface Morphology

**Figure 9:**
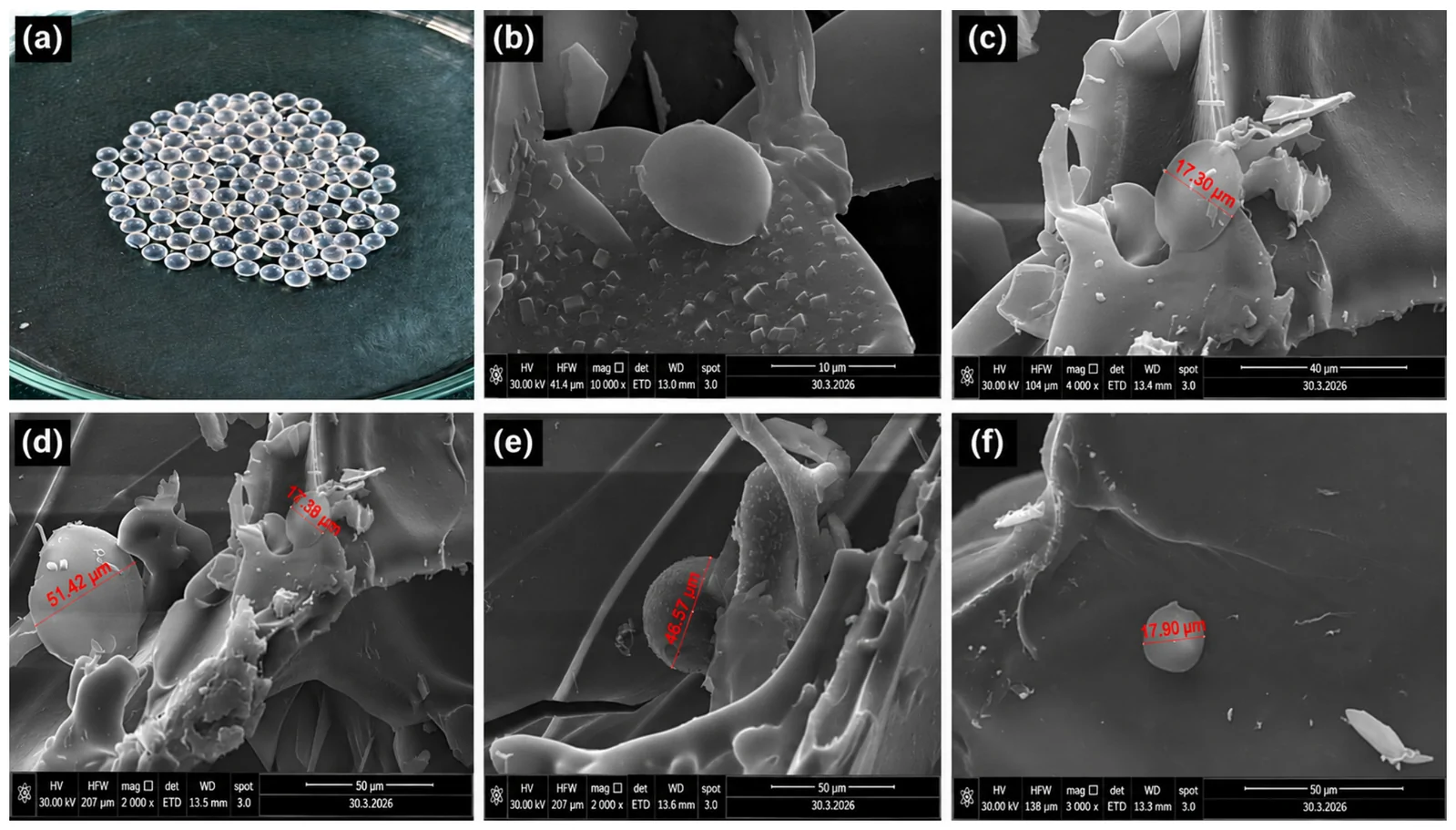
Appearance (**a**) and scanning electron microscopy (SEM) micrographs of SAlg/WP–MAPE hydrogel beads (**b**–**f**).

The surface morphology and microstructure of SAlg/WP–MAPE beads were evaluated using SEM, as presented in Figure 9. The beads exhibited relatively uniform spherical to oval-shaped morphology with dense and continuous surfaces, indicating successful formation of a stable encapsulation matrix through ionic gelation among sodium alginate, whey protein, and calcium ions. Similar morphologies have been reported in alginate–protein encapsulation systems containing phenolic compounds. Slight surface shrinkage, folding, and wrinkling were observed after freeze-drying, which may be attributed to ice crystal formation and water sublimation during lyophilization, resulting in partial surface collapse and polymer contraction [25]. The wrinkled morphology may additionally contribute to improved encapsulation performance by limiting oxygen diffusion and reducing premature leakage of phenolic compounds, thereby enhancing the oxidative stability of the encapsulated bioactive compounds [56]. Moreover, the absence of major pores or fractures suggests the formation of an integrated and compact barrier structure capable of efficiently entrapping phenolic compounds within the hydrogel network. The observed particle size range (approximately 17–51 μm) confirmed the formation of microscale particles with relatively homogeneous distribution, which may facilitate improved dispersion within the yoghurt matrix and contribute to controlled release of encapsulated phenols. In addition, incorporation of whey protein may enhance matrix cohesion and structural stability through protein–polysaccharide interactions and hydrogen bonding, which is consistent with the FTIR results obtained in the present study [55].

### 3.8. Characterization of Fortified Yoghurt

#### 3.8.1. Color Parameters

**Figure 10:**
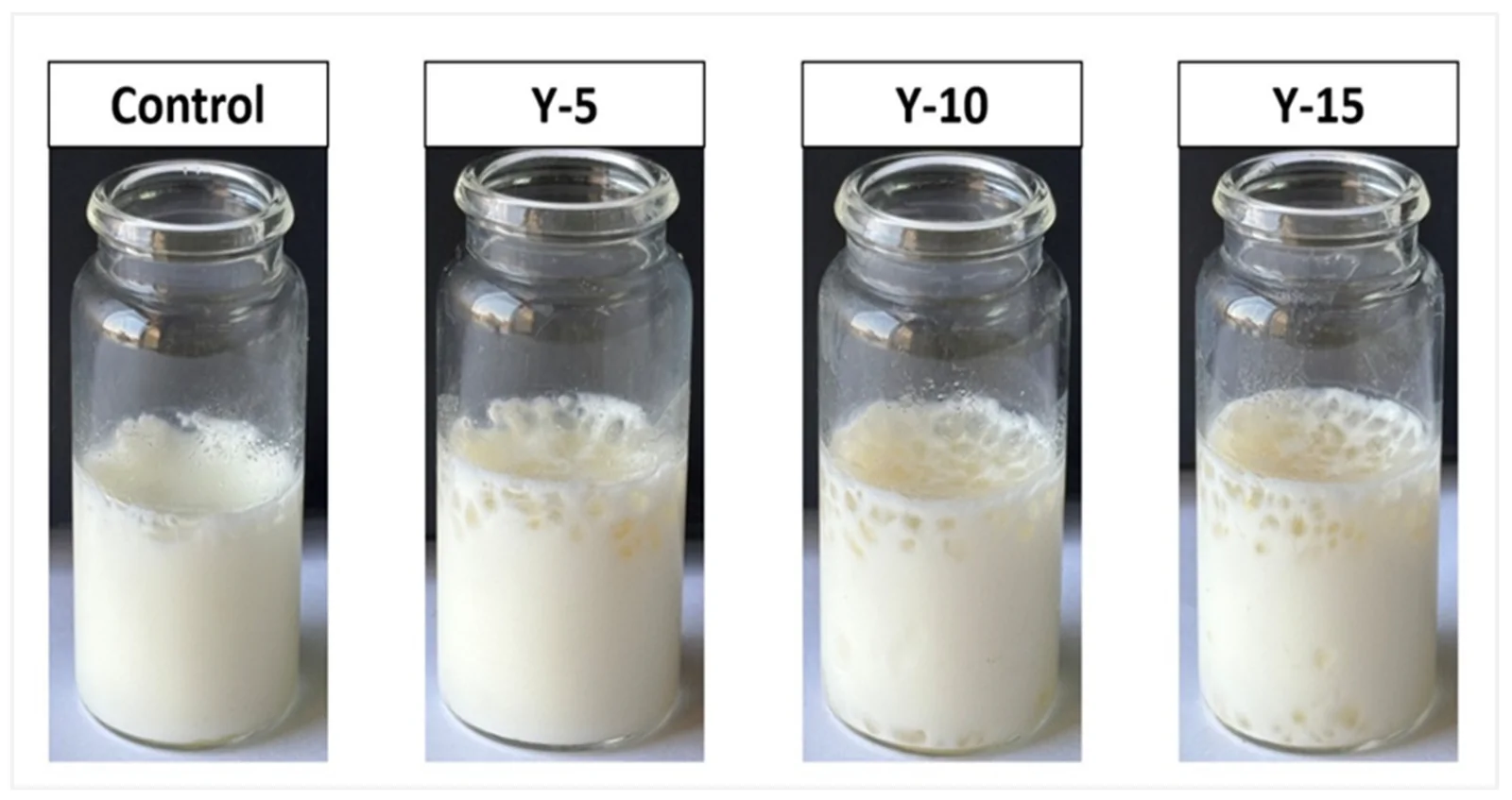
Visual appearance of yoghurt fortified with 0%, 5%, 10%, and 15% (*w*/*w*) SAlg/WP–MAPE hydrogel beads.

The visual appearance of yoghurt samples fortified with different concentrations of SAlg/WP–MAPE is presented in Figure 10, and the corresponding color parameters (L*, a*, b*, C*, and ΔE) are summarized in Table 4. All yoghurt samples exhibited homogeneous coagulation without visible sedimentation or phase separation, indicating that the SAlg/WP encapsulation system effectively stabilized MAPE within the yoghurt matrix during fermentation and refrigerated storage. Fortification progressively altered yoghurt color from bright white to creamy yellow tones with increasing concentrations of encapsulated MAPE. These changes are mainly associated with the natural pigments and phenolic constituents present in the extract, as well as the protective effect of encapsulation against oxidation and degradation of bioactive compounds during processing.

Fortification significantly affected all color parameters of yoghurt samples (*p* < 0.05) (Table 4). The control yoghurt exhibited the highest L* values, whereas the fortified samples showed progressively lower L* values as the SAlg/WP–MAPE concentration increased. The reduction in lightness may be related to the incorporation of phenol-rich encapsulated particles and increased total solids within the yoghurt matrix, which reduced light reflection and modified light-scattering behavior. In contrast, the a*, b*, and C* values increased significantly with increasing fortification level, indicating enhanced red-yellow color intensity and greater color saturation in fortified yoghurt samples. In addition, the ΔE values of all fortified treatments exceeded the visual perception threshold (ΔE > 3), confirming noticeable color differences compared with the control yoghurt. Similar color modifications were previously reported in yoghurts fortified with encapsulated plant phenolics and natural antioxidants [25]. Importantly, the observed color changes may positively influence consumer perception by enhancing yoghurt’s visual identity as a naturally fortified functional product.

**Figure 11:**
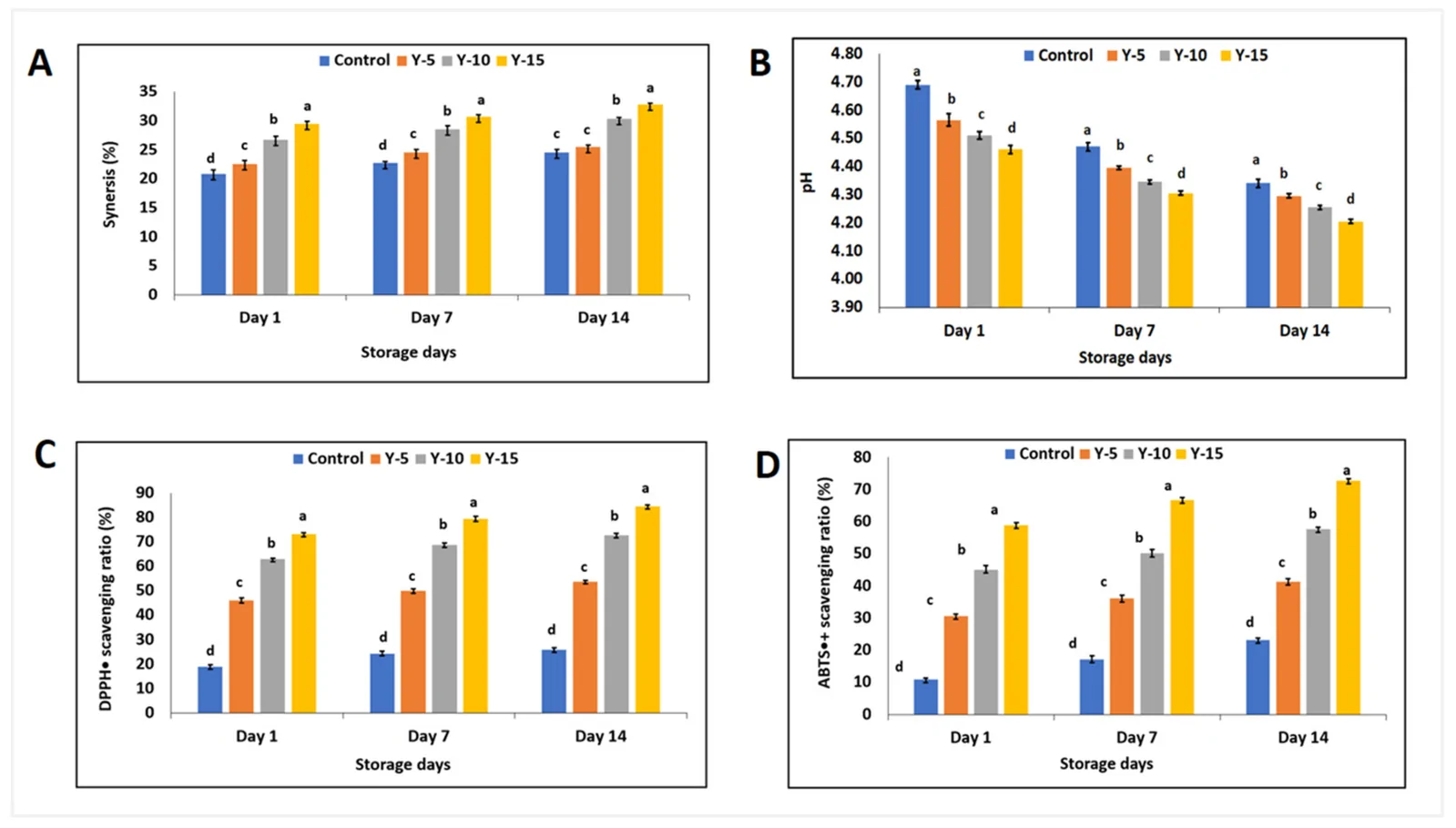
Changes in (**A**) syneresis, (**B**) pH, (**C**) DPPH^•^ radical scavenging activity, and (**D**) ABTS^•+^ radical scavenging activity of yoghurt during refrigerated storage at 4 °C for 1, 3, 5, and 7 days. Different letters within the same histogram indicate significant differences (*p* < 0.05).

The effects of SAlg/WP–MAPE fortification on syneresis, pH, and antioxidant activity of yoghurt during 14 days of refrigerated storage are presented in Figure 11. Syneresis values increased significantly (*p* < 0.05) throughout storage in all treatments, with fortified yoghurt samples exhibiting higher whey separation than the control treatment (Figure 11A).

#### 3.8.2. Changes in Syneresis, pH, and Antioxidant Activity in Different Prepared Yoghurt Samples

The progressive increase in syneresis during storage is consistent with rearrangement and contraction of the casein gel network during post-acidification, resulting in gradual whey expulsion. Moreover, the extent of this effect appeared to increase with increasing SAlg/WP–MAPE incorporation, as reflected by the progressively higher syneresis observed from Y-5 to Y-10 and Y-15. At higher MAPE loading, the greater amount of encapsulated phenolics introduced into the yoghurt matrix may provide more opportunities for interactions with casein molecules, interfering with protein–protein associations involved in maintaining the continuous gel network. Such interactions may reduce the ability of the casein matrix to immobilize water and promote localized network discontinuities, thereby facilitating whey migration from the gel. Consequently, the higher MAPE loading in Y-15 may have produced a greater perturbation of the gel structure than Y-5, resulting in increased whey separation. Similar structural modifications leading to increased syneresis were previously observed in yoghurts fortified with encapsulated polyphenols [25]. Nevertheless, the obtained syneresis values remained within the acceptable range reported for functional yoghurt products.

As shown in Figure 11B, pH values decreased gradually during refrigerated storage across all treatments, reflecting the normal post-acidification behavior of yoghurt cultures. Fortified yoghurt samples exhibited lower pH values than the control, which may be attributed to the acidic nature of MAPE and to continuous lactic acid production by starter cultures during storage. Furthermore, phenolic compounds may influence bacterial metabolic activity and fermentation kinetics through interactions with microbial enzymes and cellular metabolism. Comparable reductions in pH during storage were previously reported in yoghurts enriched with encapsulated plant phenolicss and bioactive compounds [25].

The antioxidant activity of yoghurt samples as determined by DPPH^•^ and ABTS^•+^ radical scavenging assays (Figure 11C,D) significantly increased following fortification with SAlg/WP–MAPE and continued to increase throughout storage. The enhanced antioxidant capacity is primarily related to the high phenolic content of MAPE, particularly flavonoids and phenolic acids identified by HPLC analysis. More importantly, the incorporation of MAPE within the SAlg/WP hydrogel system was associated with sustained antioxidant activity of the fortified yoghurt throughout refrigerated storage, suggesting that the developed delivery system effectively retained the functional properties of the encapsulated phenolics within the yoghurt matrix. This controlled release behavior may explain the sustained increase in antioxidant activity observed during storage.

In addition to phenolic compounds, proteolytic activity of yoghurt starter cultures during storage may contribute to the release of antioxidant peptides from milk proteins, further enhancing the antioxidant capacity of fortified yoghurt. Moreover, synergistic interactions between released bioactive peptides and encapsulated phenols may have amplified radical scavenging activity in fortified treatments [60]. These findings demonstrate that sodium alginate–whey protein encapsulation effectively preserved the functionality of *M. argun* phenolics, enabling their successful incorporation into yoghurt systems and improving antioxidant stability and functional properties during refrigerated storage.

## 4. Conclusions

This study demonstrated that *M. argun* phenolic extract (MAPE) is a promising source of multifunctional bioactive compounds rich in flavonoids and phenolic acids with considerable antioxidant, antimicrobial, and moderate antiviral activities. Molecular docking analysis further supported the potential contribution of rutin, chlorogenic acid, and rosmarinic acid to HAV protease inhibition. Encapsulation of MAPE within sodium alginate–whey protein (SAlg/WP) hydrogel beads achieved high encapsulation efficiency and successfully formed a stable polymeric network, as confirmed by FTIR and SEM analyses. Intermolecular interactions among alginate, whey protein, and phenolic compounds improved phenolics retention and the encapsulation system’s structural stability. Incorporation of encapsulated MAPE into yoghurt significantly enhanced antioxidant activity during cold storage while maintaining acceptable physicochemical characteristics and gel stability. The SAlg/WP delivery system effectively protected phenolic compounds against degradation and enabled their successful incorporation into the dairy matrix. One limitation of the current study is the lack of sensory evaluation, which hindered our ability to assess consumer acceptability and overall palatability of the MAPE-enriched yogurt. Future research should include sensory evaluations with trained panels or consumer groups to assess attributes like appearance, aroma, flavor, texture, and acceptability, along with consumer preferences and purchase intentions for better insights into the yogurt’s sensory performance. Overall, the findings highlight the potential of alginate–whey protein hydrogel systems as efficient carriers for plant phenolics and demonstrate the feasibility of developing functional yoghurt enriched with naturally derived bioactive compounds. To the best of our knowledge, this is the first study reporting the encapsulation of *M. argun* phenolics within SAlg/WP hydrogel beads and their application in functional yoghurt systems.

## Figures and Tables

**Table 1:** Antimicrobial activity of MAPE against selected microorganisms, as determined by the broth dilution method.

Microorganism	Sample Concentrations (50 µg/mL)
*Bacillus cereus*	68.28 ± 0.42
*Staphylococcus aureus*	40.77 ± 0.49
*Bacillus subtilis*	79.03 ± 0.55
*Candida albicans*	71.60 ± 1.09
*Aspergillus niger*	44.70 ± 0.69
*E. coli*	33.91 ± 0.99
*Pseudomonas aeruginosa*	47.82 ± 0.17

**Table 2:** Minimum inhibitory concentration (MIC) and concentration-dependent inhibitory activity of MAPE against selected microorganisms, as determined by the broth dilution method.

	Inhibition of Microbial Growth (%)						
MAPE (µg/mL)	*B. cereus*	*S. aureus*	*B. subtilis*	*C. albicans*	*A. niger*	*E. coli*	*P. aeruginosa*
**25**	28.66 ± 0.84	18.36 ± 0.85	23.51 ± 0.1	43.85 ± 0.15	13.25 ± 0.63	8.36 ± 0.16	17.13 ± 0.12
**50**	56.27 ± 0.42	40.77 ± 0.49	79.03 ± 0.55	71.60 ± 1.09	44.70 ± 0.69	19.36 ± 0.89	47.82 ± 0.17
**100**	68.28 ± 0.42	52.39 ± 1.10	92.23 ± 0.68	85.69 ± 0.93	60.53 ± 0.69	25.31 ± 0.95	56.32 ± 0.52
**150**	40.95 ± 0.33	38.26 ± 0.65	88.14 ± 0.68	70.45 ± 0.52	40.62 ± 0.22	14.25 ± 0.1	38.62 ± 0.41
**200**	38.30 ± 0.65	29.54 ± 0.44	32.51 ± 0.96	22.10 ± 0.51	28.63 ± 1.06	10.36 ± 0.85	27.16 ± 0.10

**Table 3:** Molecular docking interaction parameters of compounds (1–18) and the co-crystallized ligand (BBL) within the active site of HAV 3C protease (2CXV).

No.	Compound	Binding Affinity (kcal/mol)	Affinity Bond Strength (kcal/mol)	Affinity Bond Length (In Ao from the Main Residue)	Amino Acids	Ligand	Interaction
**1**	Gallic acid	−4.34	−2.5	2.78	Lys 50	O 12	H-donor
**2**	Chlorogenic acid	−6.37	−2.8	3.04	Ala 45	O 16	H-donor
			−0.7	3.39	Asn 148	O 18	H-donor
			−0.7	3.35	Ala 45	O 15	H-acceptor
**3**	Catechin	−5.17	−1.3	3.01	Val 144	O 21	H-donor
**5**	Caffeic acid	−4.43	−2.2	2.86	Val 28	O 7	H-donor
			−3.2	2.95	Val 144	O 18	H-donor
			−1.2	2.88	Val 144	O 20	H-donor
**6**	Syringic acid	−4.29	−3.9	2.72	Lys 146	O 23	H-donor
			−2.9	2.97	Arg 26	O 10	H-acceptor
**7**	Rutin	−7.29	−1.1	3.16	Val 28	O 45	H-donor
			−1.1	3.3	Asn 148	O 51	H-donor
			−1.4	3.29	Lys 146	O 13	H-acceptor
			−0.7	3.01	Gly 170	O 45	H-acceptor
			−5.4	2.89	His 44	O 47	H-acceptor
			−0.6	2.84	Arg 26	O 51	H-acceptor
			−2.5	3.09	Arg 26	O 53	H-acceptor
**8**	Ellagic acid	−5.33	−2.2	2.74	Asn 148	O 9	H-donor
			−3	3.2	His 44	O 1	H-acceptor
			−0.6	4.41	Lys 146	6-ring	pi-H
**9**	Coumaric acid	−4.58	−5.6	2.88	Ala 45	O 19	H-donor
			−0.7	4.62	Gly 19	6-ring	pi-H
**10**	Vanillin	−4.36	−3.9	2.82	Lys 146	O 1	H-donor
			−1.8	3.24	Arg 26	O 3	H-acceptor
**11**	Ferulic acid	−4.75	−1.9	2.76	His 145	O 6	H-acceptor
**12**	Naringenin	−5.02	−1.9	3.05	Lys 146	O 15	H-donor
**13**	Rosmarinic acid	−6.04	−3.7	2.87	Glu 49	O 41	H-donor
			−0.7	3.23	Arg 26	O 7	H-acceptor
			−0.7	4.83	Lys 146	6-ring	pi-H
**15**	Quercetin	−4.89	−3.2	2.98	Ser 24	O 23	H-donor
**16**	Cinnamic acid	−4.21	−0.7	3.49	Lys 146	O 18	H-donor
			−1.2	3.92	Asn 148	6-ring	pi-H
**17**	Kaempferol	−4.35	−1.5	3.31	Lys 146	O 9	H-donor
			−0.7	3.41	Asn 148	6-ring	pi-H
**19**	BBL	−4.74	−3.1	3.02	Ser 24	O 11	H-donor
			−0.8	3.31	Asn 148	O 14	H-acceptor

**Table 4:** Color parameters (L*, a*, b*, C*, and ΔE) of yoghurt fortified with different concentrations of SAlg/WP–MAPE hydrogel beads.

Yoghurt Sample	Color Parameter				
	L*	a*	b*	C*	ΔE
**Control**	75.52 ± 0.57 ^a^	−0.81 ± 0.06 ^d^	3.53 ± 0.04 ^d^	3.62 ± 0.06 ^d^	-
**Y-5**	73.48 ± 0.46 ^b^	0.69 ± 0.04 ^c^	5.21 ± 0.07 ^c^	5.26 ± 0.08 ^c^	3.05 ± 0.36 ^c^
**Y-10**	72.44 ± 0.62 ^b^	1.00 ± 0.07 ^b^	6.88 ± 0.15 ^b^	6.95 ± 0.13 ^b^	4.91 ± 0.31 ^b^
**Y-15**	70.54 ± 0.54 ^c^	1.17 ± 0.03 ^a^	8.06 ± 0.05 ^a^	8.14 ± 0.04 ^a^	7.03 ± 0.36 ^a^

All values are the means ± standard deviation of three repeated tests. Different letters in the same column (a–d) indicate significant differences (*p* < 0.05). Control, Y-5, Y-10, and Y-15 stand for the fortified yoghurt with 0%, 5%, 10% or 15% (*w*/*w*) SAlg/WP- MAPE added, respectively.

## Data Availability

The original contributions presented in this study are included in the article. Further inquiries can be directed to the corresponding author.
